# Additive-Free Commercial Alumina Catalyzes the Halogen Exchange Reaction of Long Alkyl Halides in Batch and in Flow Processes

**DOI:** 10.1021/acsorginorgau.4c00039

**Published:** 2024-08-02

**Authors:** Paloma Mingueza-Verdejo, Susi Hervàs-Arnandis, Judit Oliver-Meseguer, Antonio Leyva-Pérez

**Affiliations:** Instituto de Tecnología Química (Universitat Politècnica de València-Agencia Estatal Consejo Superior de Investigaciones Científicas), Avda. de los Naranjos s/n, 46022 València, Spain

**Keywords:** alumina, heterogeneous catalysis, inflow synthesis, halogen exchange reaction, alkyl halides

## Abstract

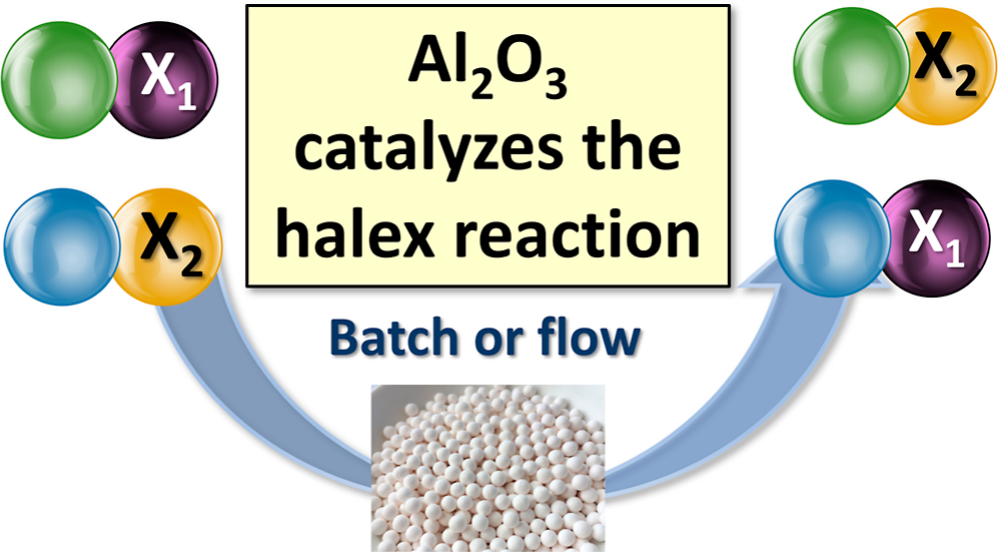

The synthesis of alkyl halides can be performed by simply halide exchange reactions between two different alkyl halides, catalyzed by aluminosilicates. Here, we show that commercially available alumina shows a superior catalytic activity for the halogen exchange reaction between long alkyl halides (more than 6 carbons), including fluorides, in either batch or flow modes. The catalytic activity of the solid alumina is modulated by alkaline countercations on the surface, and sodium-supported alumina shows the optimal performance for the iodo-bromo and iodo-fluoro exchange under inflow reaction conditions, after >24 h reaction time, without any external additive.

## 1 Introduction

**Figure 1:**
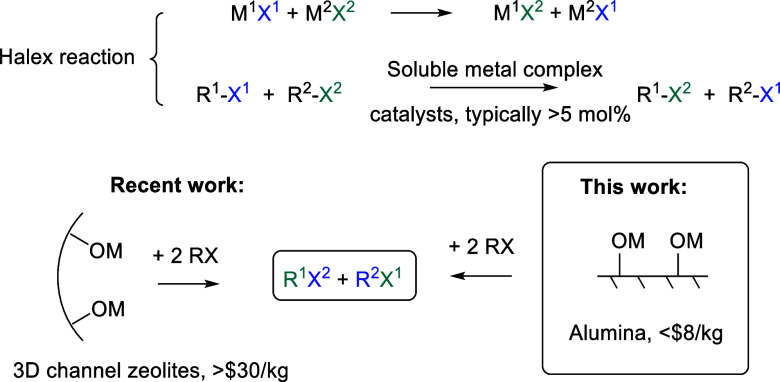
Halogen exchange (halex) reaction. M, M^1^, M^2^: protons or different metal cations; X^1^, X^2^: I, Br, Cl, or F; R, R^1^, R^2^: long alkyl chains.

Alkyl halides are fundamental building blocks in organic synthesis.^1^ Particularly, iodo and bromo derivatives act as convenient chemical handles to allow for the easy introduction of functional groups in organic molecules. The preparation of alkyl iodide from alkyl bromide or chloride with potassium or sodium iodide in acetone was initially reported by Finkelstein in 1910.^2^ However, the use of solvents, as for example dimethylformamide or other cocatalyst, such as CuI, made this procedure environmentally inviable. Therefore, from a sustainable point of view, the synthesis of alkyl halides from other preformed and widely available organic halides is of interest. In fact, the chemical industry has exerted a great effort in the optimization of this reaction for industrial applications.^3−6^ This transformation, called halogen exchange (halex) reaction, and shown in Figure 1, is well-known for metal salts and aromatic halides, where the participation of catalytic or stoichiometric amounts of metals is necessary.^7−15^ In contrast, the halex reaction of alkyl halides is much less developed and usually requires the use of expensive metal catalysts.^16−21^ Despite this reaction probably not being used in the chemical industry yet, finding an inexpensive and sustainable catalyst could be of interest to achieve a wider implementation of this reaction.

We have recently reported that zeolites catalyze the halex reaction of a variety of iodo-, bromo- and chloro-alkyl derivatives in good yield and selectivity, without the addition of any solvent or additive.^22^ The countercation of the zeolite plays a key role during the catalysis since the nature of M (see Figure 1) is essential to achieve an optimal catalytic performance.^23^ These aluminosilicate solids are able to perform the halex reaction not only in batch but also in flow mode; however, the microporous structure of the zeolite somewhat hampers the diffusion of long alkyl molecules through the catalytic framework, and the pores become blocked after 3 h reaction time.^22^ Therefore, at this point, we considered the possibility of using alumina as an open surface solid catalyst to enable the molecular traffic on the surface while keeping a good catalytic activity for the halex reaction.^24^ We show here that simple commercial alumina can do the catalytic process, even for a long alkyl fluoride.^5^

Alumina was reported, more than 20 years ago, to catalyze the halide disproportionation reaction of freons (CH_*x*_Cl_*y*_F_*z*_; *x*–*z* = 0–2)^25^ and the halex reaction with gaseous alkyl derivatives (mainly up to four carbon atoms),^26^ only when the alumina surface was modified with fluoride atoms at high temperatures (up to 500 °C)^27^ or with tetrabutylphosphonium bromide,^26^ respectively. The latter was also able to catalyze the reaction in a flow mode. However, as far as we know, recent studies on alumina-catalyzed halex reactions have not been reported, particularly in the halex reaction of long alkyl derivatives.^28^ In the modern context of sustainable organic synthesis, the use of alumina as a catalyst for organic halides is of relevance. In contrast to zeolites, alumina is commercially available in different cationic forms (from protons to calcium), with prices around $8 per kilogram, while most cation-exchanged zeolites must be often prepared, or in any case, they are commercially available at prices higher than $30 per kilogram. Thus, the use of alumina for the inflow halex reaction of alkyl halides has advantages over zeolites not only from the technical point of view (better diffusion and commercial availability of different cation-exchanged alumina samples) but also from the economic side.

## 2 Experimental Section

### 2.1 General Preparation of Cation-Exchanged Alumina Samples

Alumina in Ca^2+^, Na^+^, and H^+^ forms, are commercially available (chromatographic grade). The rest of the alumina samples were prepared after treating Na^+^–Al_2_O_3_ with a 0.1 M aqueous solution of LiOAc, KOAc, or CsOAc, respectively, at 70 °C for 24 h, to give the corresponding Li^+^–Al_2_O_3_, K^+^–Al_2_O_3_, and Cs^+^–Al_2_O_3_ materials after vacuum filtration and extensive washings with distilled water. The extent of metal incorporation was assessed by inductively coupled plasma–optical emission spectroscopy (ICP–OES, see Table S1 in the Supporting Information).

### 2.2 General Reaction Procedure in Batch Mode


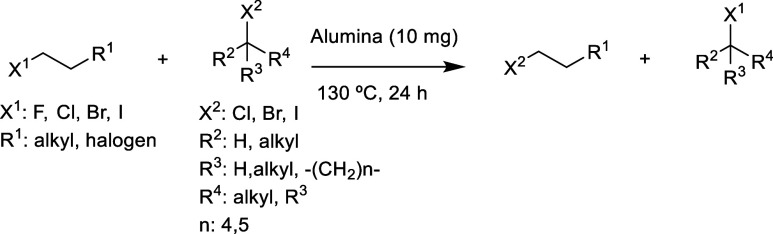
Reagents (1 mmol and 5–10 equiv, respectively) were introduced in 6–7 mL sealed vials containing a magnetic stirrer and the alumina catalyst (10 mg, unless otherwise indicated). The mixture was allowed to react for 1–3 days in a preheated oil bath at 130 °C under atmospheric pressure. Aliquots (typically 25 μL) were periodically taken from the supernatant, and gas chromatography (GC) samples were prepared after dilution of the reaction mixture in a vial with 1 mL of dichloromethane (DCM) and *n*-dodecane as an external standard. Products were characterized by GC–mass spectrometry (GC–MS) and nuclear magnetic resonance (NMR) and compared with existing literature when available.

### 2.3 Typical Reaction Procedure in Flow Mode

1,8-Dibromooctane **1** (35 mmol) and 1-iodobutane **2** (5 equiv) were placed in a 50.0 mL syringe. The mixture was pumped in countergravity mode at atmospheric pressure, and at a flow of 0.1 mL·min^–1^, on the bottom of a stainless-steel tube with a 1 cm internal diameter and filled with chromatographic grade pelletized Na^+^–Al_2_O_3_ (sieved to a particle size of 0.4–0.8 μm).

#### 2.3.1 Procedure 1

The tube was filled entirely with 10 g of pelletized Na^+^–Al_2_O_3_. The reaction took place at 130 °C, and samples were collected by gravity after passing through a *U*-tube. The samples were analyzed by GC after dilution of the reaction mixture in a vial with 1 mL of DCM and *n*-dodecane as an external standard.

#### 2.3.2 Procedure 2

The tube was filled with 350 mg of pelletized Na^+^–Al_2_O_3_, and 17.6 g of SiC (>1.2 μm) was added to favor the uniform distribution of the flow through the catalyst bed and thus avoid preferential pathways. The reaction took place at 130 °C, and samples were collected by gravity after passing through a *U*-tube. The samples were analyzed by GC after dilution of the reaction mixture in a vial with 1 mL of DCM and *n*-dodecane as an external standard.

## 3 Results and Discussion

**Figure 2:**
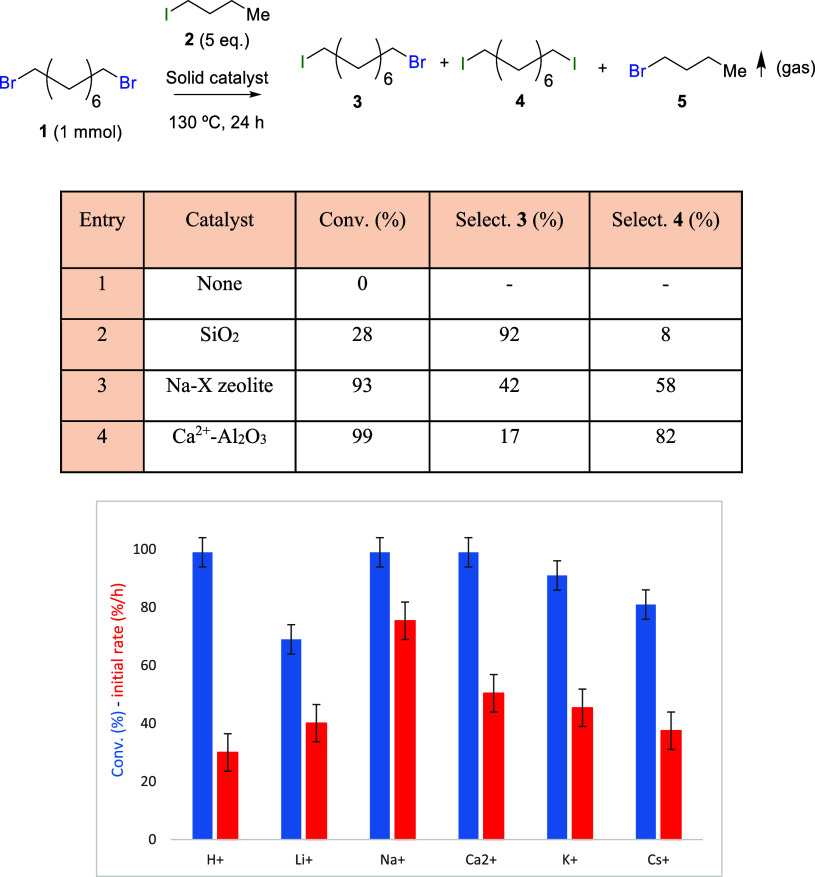
Top: catalytic results for the halex reaction between dibromooctane **1** and iodobutane **2** obtained with different solid catalysts (GC yields). Products were characterized by GC–MS and NMR. Reaction conditions: 1,8-dibromooctane **1** (1 mmol), 1-iodobutane **2** (10 mmol), 10 mg of solid catalyst (3.5 wt % respect to **1**), ambient atmosphere, 130 °C, 24 h. Bottom: conversion (blue) and initial rate (red) results for the halex reaction between dibromooctane **1** and iodobutane **2** with alumina catalysts having different surface countercations. Initial rates are calculated from the slope of the initial points after linear regression (up to 15 min reaction time and 20% conversion). Error bars account for 5% of the uncertainty.

Figure 2 shows the catalytic results for the cheapest commercially available alumina sample (Ca^2+^–Al_2_O_3_, $4/kilogram), which is basic since it holds Ca^2+^ cations on the surface. Qualitative quenching analysis with phenolphthalein confirmed the basic pH in water of the alumina. The halex reaction of 1,8-dibromooctane **1** with an excess of iodobutane **2**, under additive-free, solventless, and ambient atmosphere reaction conditions, was chosen as a model reaction. Mono- and di-iodinated octane products **3** and **4** are obtained together with bromobutane **5**, which is expelled as a gas and helps to shift the equilibrium toward the desired iodinated products, despite the main driving force of the reaction being the use of an excess of iodinated compound (see ahead). Iodobutane **2** is among the cheapest organic iodides (according to different commercial houses, its price is $0.3/gram, compared to $0.5/gram for iodoethane and $0.6/gram for iodobenzene); thus, the impact in materials cost for the reaction is minimized and acceptable, considering that bromides, chlorides, and fluorides are much cheaper. The obtained iodide products have therefore higher added value.

The results in Figure 2 show that the reaction does not proceed without any catalyst under the indicated reaction conditions after 24 h reaction time (entry 1), and that SiO_2_ barely catalyzes the reaction (28% conversion, entry 2), and that Na–X zeolite acts as an efficient solid catalyst (93% conversion, entry 3),^22^ however, without marked selectivity for products **3** or **4** (ca. 4:6 molar ratio). In contrast, Ca^2+^–Al_2_O_3_ acts as a superior catalyst, giving 99% conversion and 82% selectivity to diiodooctane product **4**. Notice here that two catalytic events occur for product **4;** thus, the number of catalytic cycles of Ca^2+^–Al_2_O_3_ is much higher than that of Na–X zeolite. The amount of solid employed in the total reaction mixture is <0.5 wt % (10 mg in ∼2 g; 3.5 wt % with respect to limiting dibromooctane **1**), which is suitable for industrial batch reactors. This result encouraged us to study other alumina samples with different surface countercations since it is precisely the countercation which plays a key role in the catalytic activity of the zeolite.^22^Figure 2 also shows the conversion and initial rates observed for alumina samples containing Ca^2+^ (commercial), Cs^+^, K^+^, Na^+^ (commercial), Li^+^, and H^+^ (commercial), in order of decreasing basicity (detailed numeric data and selectivity can be found in Table S1 in the Supporting Information). The noncommercial samples were prepared by an aqueous cationic exchange procedure with the corresponding acetate salts and the commercial Na^+^–Al_2_O_3_, akin to the preparation of cation-exchanged zeolites,^29^ and the amount of cation introduced was assessed by ICP–OES (typically 0.1–10 mmol of metal cation/gram of alumina, according to ICP–OES, see Table S1). The samples were characterized by X-ray diffraction (XRD), and the alumina was not modified, except for the Cs^+^ exchange (see Figure S1). The catalytic results show that the conversion is high for most of the alumina samples; however, the initial rate is maximum for commercial Na^+^–Al_2_O_3_, with a value of 75.6 h^–1^. In other words, the Na^+^-containing alumina converts all the dibromide **1** in less than 2 h, with high selectivity for the diiodooctane **4** at extended reaction times (83% of **4** after 24 h, see Table S1). It can be seen that the samples obtained by ion exchange have a lower conversion percentage performance compared to the pristine commercial samples, and we attribute the lower performance of the former to a somewhat ineffective ion exchange since the higher amounts of cations correspond to higher conversions (10.1 mmol/g K^+^ has 91% conversion and 4.96 mmol/g Li^+^ has 69% conversion). The mass balance of the reaction was complete according to the isolated yields (several reproductions) and the GC analyses with *n*-dodecane as an external standard. Thus, we choose Na^+^–Al_2_O_3_ for the leaching, reusability, scope, and inflow studies. It is true that H^+^–Al_2_O_3_ is more active in the long term than Na^+^–Al_2_O_3_, but the latter is faster, and H^+^–Al_2_O_3_ could be more reactive toward other functional groups by the inherent acidity of the material. Please notice here that no selectivity issues are associated with the cations, just catalytic activity (product **4** comes from **3**).

With these results in hand, we first proceeded to study the scope of the Na^+^–Al_2_O_3_ catalyst for the halogen exchange reaction with alkyl derivatives, and the results are shown in Table 1. It can be seen that generally good yields are obtained for a variety of alkyl chains, for primary and secondary iodo-bromo exchanges (entries 1, 2, and 6). The use of a lower boiling point iodide such as iodoethane **6** (entry 2) also gave a good yield of (bis)iodinated product **4** (∼82%) and allowed a better isolation of the corresponding products after evaporation of the reactant in excess (see Supporting Information). However, it must be noted here that the substitution of one halogen for another does not change the boiling point or the polarity of the compound that much to enable the separation of them either by distillation or silica chromatography, respectively. For that reason, we only were able to isolate here the products when the reaction is totally complete and, in addition, one of the starting materials can be eliminated under vacuum. For Cl–I, F–I, and F–Br (entries 3–5), the results are also good. However, when one of the reactants is a cycloalkyl, the conversions decrease (entries 7–12), even if the halogen is in the alkyl chain (entry 8). However, when one of the reactants is a cycloalkyl, the conversions decrease (entries 7–12), even if the halogen is in the alkyl chain (entry 8), bromo- and chloro-alkyl derivatives are obtained. Remarkably, a fluoride alkyl compound (fluorooctane, **9**) could be transformed into the iodide and bromide counterpart, **8** and **11**, respectively, in reasonably good yields (entries 4 and 5) and moderate yields (entry 8). Unfortunately, perfluorodecalin and perfluorooctanoic acid did not react under the optimized reaction conditions, although a further exploration of the reaction conditions could lead to a general defluorination reaction, which will be studied in due course. It is true that an excess of alkyl iodide increases the price of the process; however, various examples show that the alkyl iodide cannot be in excess (entries 9–12, and entries 1 and 2 in Table 2 ahead). The reaction was also performed at a higher scale to see if the same yield was obtained. For this, we tested a reaction between **1** and **2** using 10 mmol of **1** instead of 1 mmol. Conversion resulted in 98.8% (selectivity for **3**: 21.5% and selectivity for **4**: 78.5%). It must be noticed here that, despite the differences in volatility or polarity between the starting material and the final product being low and because of this it could be difficult to purify the reaction products obtained, the fact that high yields of alkyl iodides are obtained in many cases avoids any separation between the starting halide and the final iodide product, and only the starting iodide (in excess), with a rather different (lower) boiling point, has to be separated and recovered. This excess of iodide, typically the case for *n*-butyl iodide, can be recovered after the low amount of *n*-butyl bromide or chloride in the recovered mixture is also separated by distillation (boiling points for these volatile substances differ significantly).

**Table 1:**
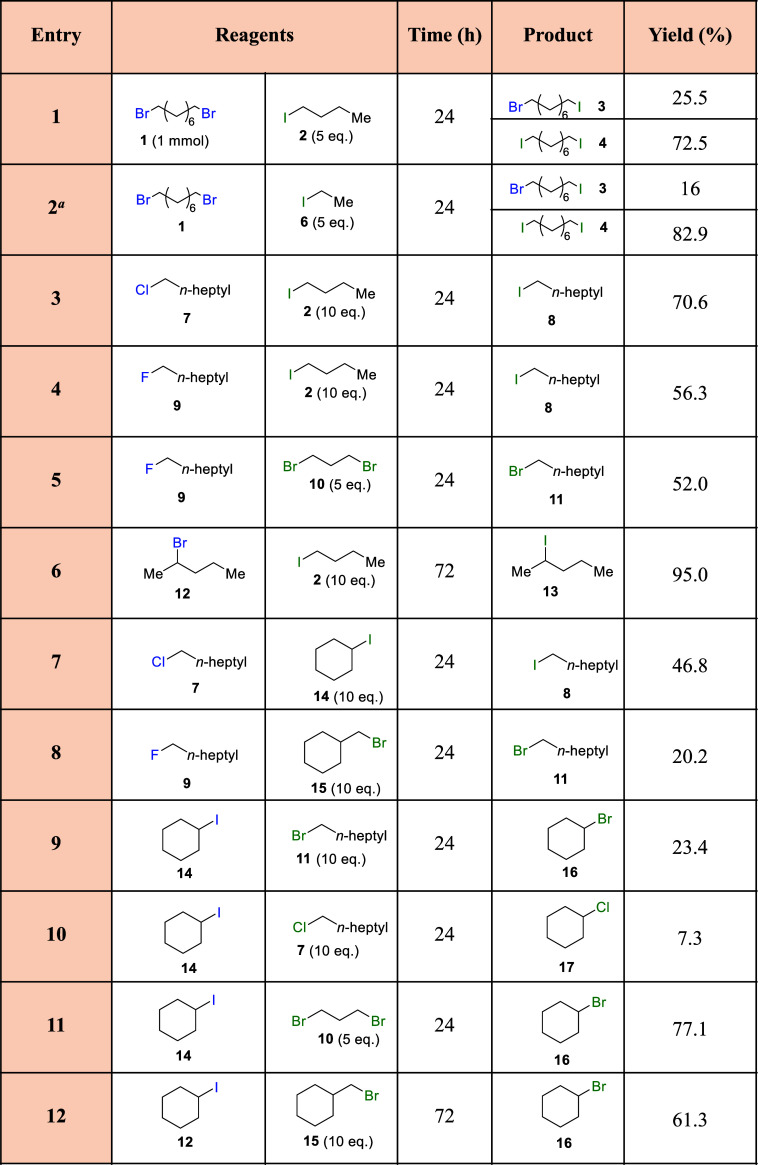
Scope Results for the Halex Reaction Catalyzed by the Na^+^–Al_2_O_3_ Catalyst^a^

a GC yields. Reaction conditions: reagent 1 (left, 1 mmol), reagent 2 (right, 10 mmol), 10 mg of Na^+^–Al_2_O_3_, ambient atmosphere, 130 °C. Products were characterized by GC–MS and compared with existing literature when available. ^a^This reaction was also performed using acid alumina under the same reaction conditions, obtaining the final products **3** and **4** in 16.2 and 83.8%, respectively.

**Table 2:**
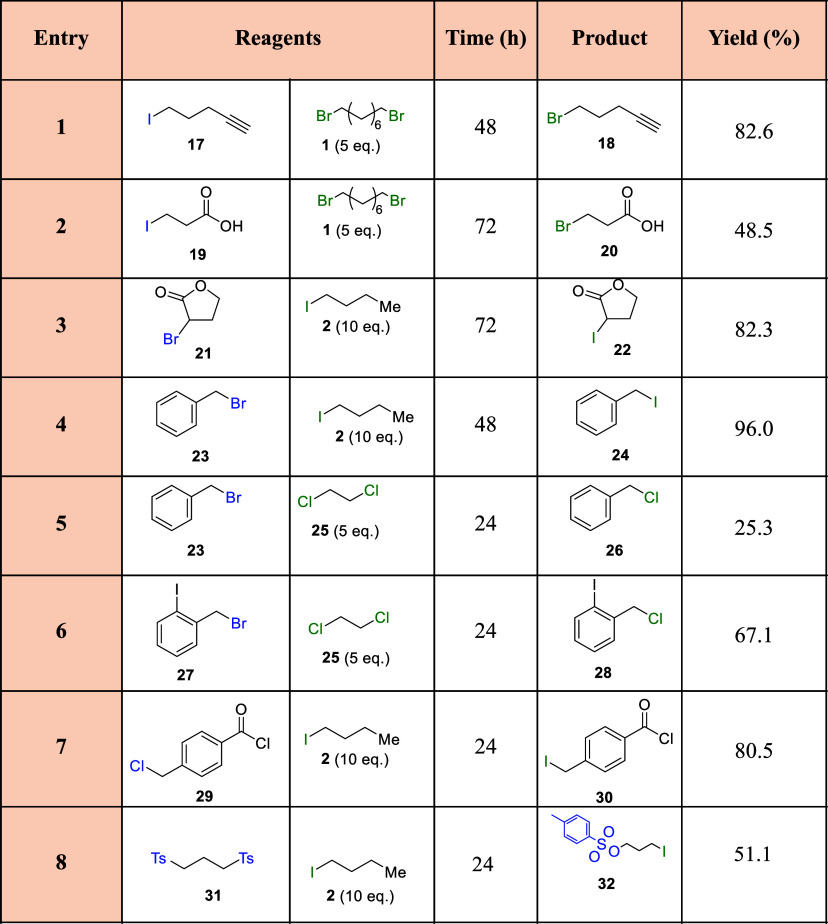
Scope Results for the Halex Reaction Catalyzed by the Na^+^–Al_2_O_3_ Catalyst in the Presence of Other Functional Groups^a^

a GC yields. Reaction conditions: reagent 1 (left, 1 mmol), reagent 2 (right, 10 mmol), 10 mg of Na^+^–Al_2_O_3_, ambient atmosphere, 130 °C. Products were characterized by GC–MS and compared with existing literature when available.

After observing the difference in the selectivity between the Br and I exchange for different iodoalkane chains, we performed kinetic experiments with different iodides, confirming that the reaction rate majorly depends on the number of carbons of the chain, i.e., the lower the number of carbons, the better the initial rate, with the exception of iodo-*n*-octane (Figure S2). The influence of the initial amount of each haloalkane was also tested, observing that higher initial amounts of the iodo derivative **2** gives faster initial rates, while same initial amounts or higher amounts of the less reactive halogen derivative dibromide **1** decreases the initial rate by 10 times (Figure S3), thus supporting that the driving force of the reaction is the use an excess of iodide reactant. In any case, regarding atom economy, the halogen atoms are not lost but distributed again between two new alkyl halides with different boiling points; thus, it is possible to have two useful products from two different starting materials with complete atom selectivity after isolation.

Moreover, we also generalize the scope to other functional groups, as shown in Table 2. With these results in hand, we can confirm that the halogen exchange between I and Br is also possible in the presence of other functional groups with good to excellent yields (entries 1–4). Additionally, we were able to perform the reaction in the presence of additional halogen atoms in aromatic position, which are not reactive (entry 6), and also in the presence of an acyl chloride (entry 7), which also did not react. Besides, pseudohalogen groups such as TsO were also exchangeable (entry 8), giving moderate yields using an excess of iodobutane **2**. In this case, we are also able to clearly observe the formation of butyl 4-methylbenzenesulfonate **33**, i.e., the other exchanged product, in the same amount as **32**.

A hot filtration test indicates that there is not any catalytic active species in solution since the halex reaction stops after removal of the solid alumina catalyst after 1 h at the reaction temperature (130 °C, Figure S4). In accordance, the Na^+^–Al_2_O_3_ solid catalyst could be reused four times with a minor decrease in conversion, from 93% in the first use to 81% in the fourth use (Figure S5, notice that the first use is here adjusted to not have complete conversion, in order to analyze the differences in conversion with subsequent reactions. With higher times, the conversion after reuses increases). The slight conversion decrease observed throughout the inbatch reuses could be attributed to a strong adsorption of halides on the catalytically active surface Lewis acid sites, in line with previous studies with freon-type molecules.^27,30,31^ The reaction mechanism involved in this process should be similar to the one proposed for zeolites,^22^ in which an Al–O–Al bond participates in the halogen scrambling between halocompounds (Figure S6). Therefore, at this point, it was envisioned that the employment of an inflow system could be beneficial for the Na^+^–Al_2_O_3_ catalyst lifetime since undesired poisoning halides could flow away in a continuous tubular reactor.

We then performed the inflow reactions. First, the Na^+^–Al_2_O_3_ catalyst was pelletized to 0.4–0.8 μm diameter size particles, and a comparative analysis of the powder and pelletized samples by XRD (Figure S7), Brunauer–Emmett–Teller surface area measurements (BET surface area, Table S2), Fourier transform infrared spectroscopy (FT-IR, Figure S8), and ^27^Al solid state magic angle spinning NMR (^27^Al ss-MAS NMR, Figure S9 and Table S3) showed a perfect matching (see Supporting Information for additional comments).^32^ Thus, we can conclude that the pelletizing process did not produce any alteration in the alumina structure.

**Figure 3:**
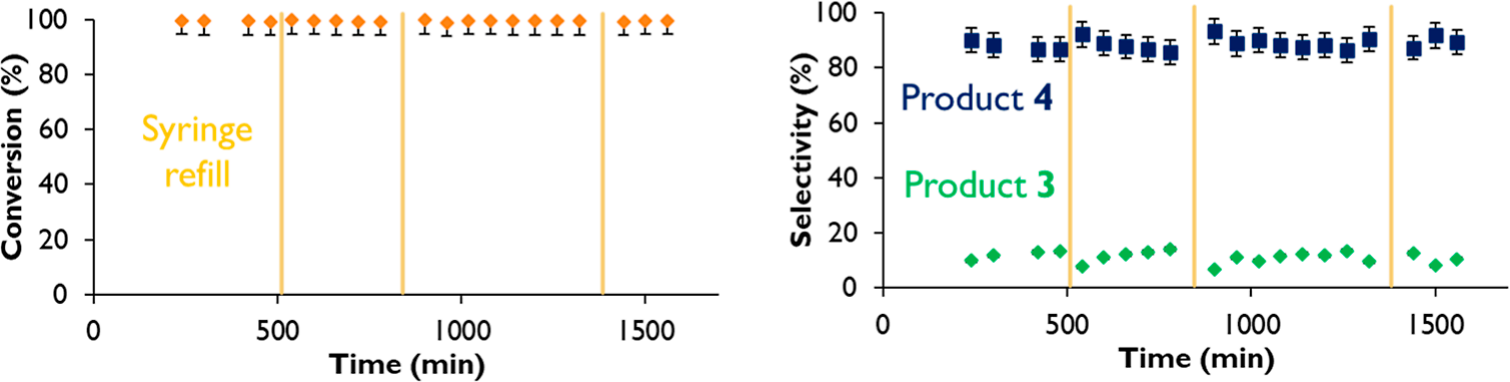
Conversion (left) and selectivity (right) results for the inflow halex reaction of **1** and **2** with 10.0 g of Na^+^–Al_2_O_3_ catalyst, pelletized between 0.4 and 0.8 μm, and placed in a fixed-bed tubular reactor (1 cm diameter, 21.5 cm length) with a reactant feed flow rate of 0.1 mL·min^–1^ at 130 °C. Error bars account for 5% uncertainty.

With the above data in hand, the inflow reaction was carried out using a tubular reactor of 1 cm diameter and 21.5 cm length (Figure S10). These dimensions allow to introduce 10 g of alumina solid catalyst to fill the reactor, occupying 2.5 mL of the reactor. That leaves 14.4 mL of free path for the reaction mixture. The liquid mixture containing **1** + **2** (5 equiv, same proportions as in the batch process) was passed through the tubular reactor in a countergravity fashion, injected by a syringe pump. A manometer was connected to the system in order to control any pressure increase after potential blocks. The flow rate was adjusted to 0.1 mL·min^–1^ at the reactor exit, and a temperature of 130 °C was measured in the middle of the tubular reactor. The catalytic results in this reactor system are listed in Figure 3.

The inflow reaction proceeds for at least 1600 min (>26 h) at >99% conversion and ∼90% selectivity for the di-iodinated product **4** without any depletion in the catalytic activity. This catalyst lifetime is four times longer than zeolite NaX (6 h).^22^ The inflow reaction was repeated with a fresh Na^+^–Al_2_O_3_ catalyst, and the result was practically the same (Figure S11) even after decreasing the flow rate to 0.075 mL·min^–1^, showcasing the reproducibility of the system. Also notice that the selectivity between compounds **3** and **4** is constant over time, with an improved selectivity compared to batch reaction (25.5 and 72.5%, respectively). Due to the more intimate contact of the reactants and the catalyst, the probability to obtain the compound exchanged twice is improved. Characterization of the spent Na^+^–Al_2_O_3_ catalyst by XRD (Figure S12), FT-IR (Figure S13), and ^27^Al ss-MAS NMR (Figure S14 and Table S3) after 26 h reaction time shows that the Na^+^–Al_2_O_3_ catalyst keeps its original structure, which is remarkable since previous studies on halide exchange reactions with alumina as a catalyst required the formation of AlF_3_ species.^27,30^

The calculation of the space-time conversion (STC) for both the inflow and batch systems was carried out (see calculations in Supporting Information). The results show that the continuous tubular reactor is 10 times more efficient than the batch reactor [STC = 0.24 vs 0.021 mol of **1**/(L·h)], which considering also the intrinsic advantages of the inflow system (absence of batch charges, washings, and discharges; extended reaction times; easy monitoring; etc.) makes the former much more efficient than the batch process. Indeed, a high weight productivity (4 g of product **4** per gram of Na^+^–Al_2_O_3_ catalyst) was obtained in flow mode after just ∼1 day (26 h) reaction time (see also Supporting Information for Figure S15a and additional comments).

We also performed the inflow experiment with functionalized bromo derivative **21**, observing that the corresponding product α-iodo-γ-butyrolactone **22** was not mainly formed, but the dehalogenated lactone **34** was obtained (Figure S15b). We presumed that the dehalogenation was produced by the water adsorbed in the alumina, and in order to confirm this, the reaction was repeated with D_2_O added on the alumina and observed the formation of **34**-*d*_**1**_ also in higher amounts than the corresponding halogenated product (Figure S15c). The deuterated product **34**-*d*_**1**_ was confirmed by GC–MS analysis (Figure S15d). It must be noticed here that the dehalogenation reaction does not proceed in the absence of the iodine compound **2**, thus the dehalogenation reaction seems to occur in the iodinated product **22** rather than in the starting bromide **21**. Indeed, when we performed the experiment with deuterated water in batch mode with only **21** and not **2** in the reaction medium, the formation of the dehalogenated product was not observed (see Experimental Section for details). The reaction mixture of α-bromo-γ-butyrolactone **21** and D_2_O gave only 2.7% of **34**. An initial analysis of the commercial α-bromo-γ-butyrolactone showed the presence of γ-butyrolactone in 1 mol %. When we repeated the experiment in the presence of BuI **2**, favoring the activation of the C–X bond, the amount of **34**-*d*_**1**_ product obtained is 87.2%, while **22** was obtained in a 12.8% yield. These results confirm that, in the presence of water, the most favored reaction is the dehalogenation of the iodo-γ-butyrolactone **22**, i.e., an iodination/dehaloprotonation cascade reaction.

**Figure 4:**
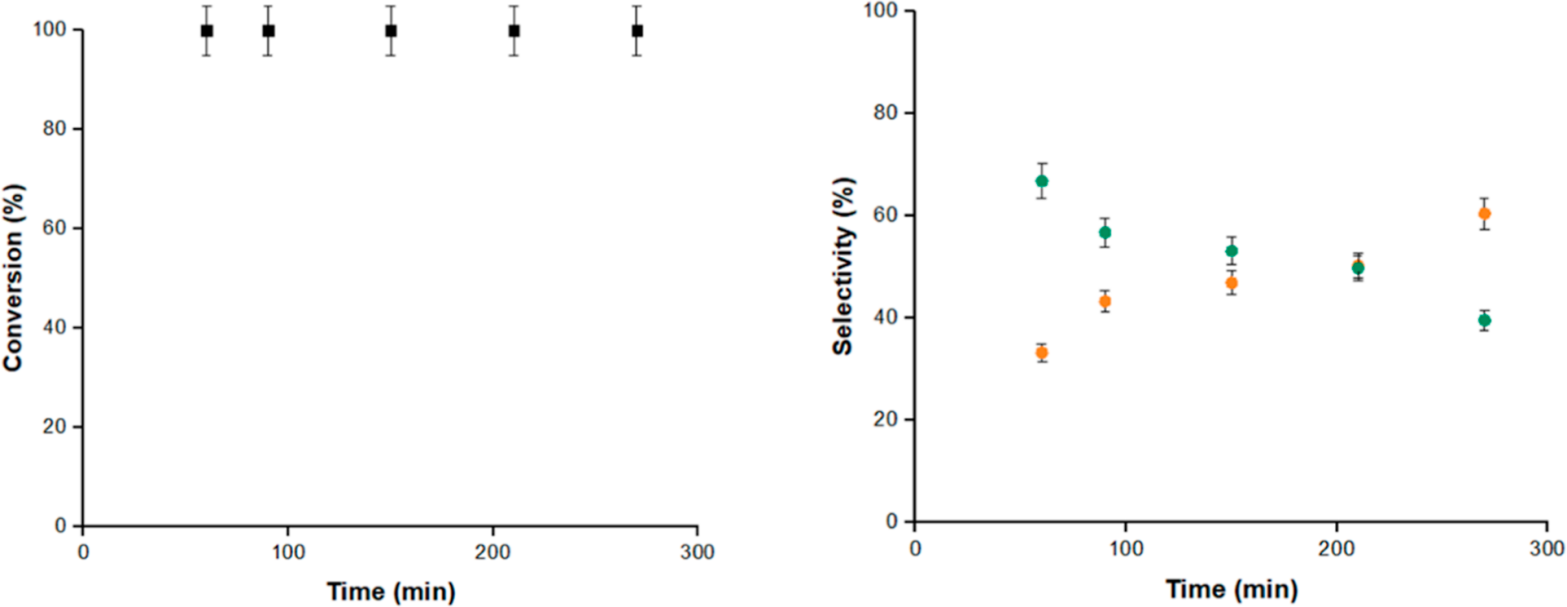
Conversion of 1-fluorooctane **9** (black points), and selectivity toward 1-iodooctane **8** (orange points) or 1-octene **35** (green points), during the inflow halex reaction of **9** and **2** with 10.0 g of Na^+^–Al_2_O_3_ catalyst (<4% of ethers were also found, not shown here). See Figure 3 for reaction conditions. Error bars account for 5% uncertainty.

The transformation^33^ and/or degradation^34^ of long-chain alkyl fluorides is a topic of high interest in environmental organic chemistry and catalysis since some long-chain alkyl (per)fluorides have been declared as persisting pollutants.^5,35^ Having in mind that 1-fluorooctane **9** is reactive under the alumina-catalyzed conditions (see Table 1, entries 4, 5, and 8, above), in contrast to zeolites where alkyl fluorides were not reactive,^22^ we performed an inflow reaction with fluoride **9** and **2**, under the same reaction conditions as with dibromide **1**. The results are shown in Figure 4, and it can be seen that complete conversion of **9** is obtained during >4 h reaction time in flow mode. 1-Iodooctane **8** is obtained in good yield, particularly after an extended reaction time in flow mode, together with the defluorination product 1-octene **35**. Minor amounts of ethers (<4%) were also found. These results strongly support the superior catalytic activity of alumina for the halex reaction compared to other metal oxides.^36^

As we performed for dibromide **1**, we also carried out here an inflow reaction with 30 times less alumina in the reactor and different flow rates, and the results (Figure S16) show that, in this case, the major product is 1-octene **35**. This result, although not good for the halex reaction, is also interesting in the context of alkyl fluoride degradation.

**Figure 5:**
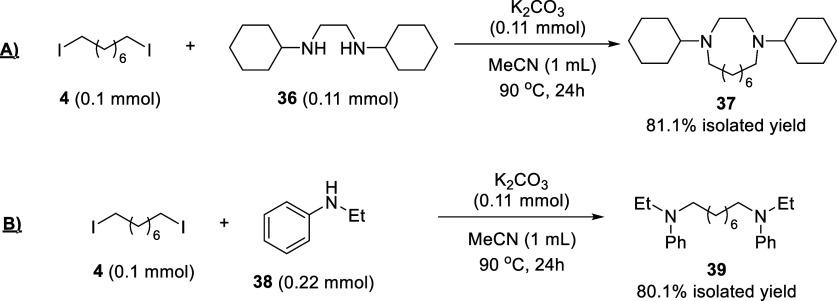
C–N coupling reactions (nucleophilic substitutions) performed with halogen product **4** obtained after the Na^+^–Al_2_O_3_-catalyzed halex reaction between **1** and **2**, without further purification.

The products obtained under either batch or flow conditions can be used, as obtained, in further C–N coupling reactions (nucleophilic substitutions), as shown in Figure 5. The resulting products **37** and **39** were obtained in high isolated yields. Notice that when we try to use dibromooctane as a starting material (as in reaction B), only one amine molecule is coupled, as confirmed by GC–MS, showcasing the superior reactivity of the diiodide intermediate product.

## 4 Conclusions

Commercially available alumina catalyzes the halex reaction of long alkyl chain iodo, bromo, chloro, and fluoro derivatives in good yields and selectivity, in both batch and inflow modes. The alumina surface countercation plays a key role for the catalytic activity, and Na^+^–Al_2_O_3_ showed the best reaction rates. This alumina catalyst can be used for more than 1 day (26 h) in flow mode without any erosion of the catalytic activity to give the halex products with complete conversion and high selectivity, including a long fluoroalkyl derivative. These results open the way to use simple, inexpensive, and widely available commercial alumina for halex reactions.

## Data Availability

The data underlying this study are available in the published article and its Supporting Information.

## Supporting Information Available

Further experimental details, including experimental techniques and calculations, catalytic results for the halex reaction between dibromooctane **1** and iodobutane **2** with different alumina samples, BET surface area results for Na^+^–Al_2_O_3_ before and after pelletizing, ^27^Al ss-MAS NMR integration results for Na^+^–Al_2_O_3_ before and after pelletizing, XRD measurements of neutral-Al_2_O_3_ and after cation exchange with K^+^, Li^+^, and Cs^+^, initial rate results depending on the length or structure of the molecule, initial rate results depending on the limiting reactant, leaching test for the halex reaction catalyzed by Na^+^–Al_2_O_3_, reuse results of the Na^+^–Al_2_O_3_ catalyst, reaction mechanism proposed for the halex reaction with alumina as a catalyst, XRD, FTIR, and ^27^Al ss-MAS results for Na^+^–Al_2_O_3_ before and after pelletizing, photograph of the in-house made tubular reactor employed in this study, conversion and selectivity results for the in-flow halex reaction of **1** and **2**, XRD, FTIR, and ^27^Al ss-MAS results for Na^+^–Al_2_O_3_ before and after reaction in the tubular reactor for 26 h reaction time, conversion results of 1-fluorooctane **9** and selectivity results towards 1-iodooctane **8**, product characterization results, and copies of NMR spectra (PDF)
