# Supplementary material for: Additive-Free Commercial Alumina Catalyzes the Halogen Exchange Reaction of Long Alkyl Halides in Batch and in Flow Processes
Source: ACS Org Inorg Au. 2024 Aug 2;4(6):640–8. doi: 10.1021/acsorginorgau.4c00039 (PMC11621953; doi:10.1021/acsorginorgau.4c00039)
Supplement: Supplementary file 1 — gg4c00039_si_001.pdf [file gg4c00039_si_001.pdf]

SUPPORTING INFORMATION

Additive-free commercial alumina catalyzes the halogen exchange reaction of long alkyl halides in batch and in flow processes.

Paloma Mingueza-Verdejo, Susi Hervàs-Arnandis, Judit Oliver-Meseguer* and Antonio Leyva-Pérez.*

Instituto de Tecnología Química (Universitat Politècnica de València- Agencia Estatal Consejo Superior de Investigaciones Científicas), Avda. de los Naranjos s/n, 46022 València, Spain.

Table of Contents

Experimental Section	SI2
General	SI2
Physicochemical techniques	SI2
Reaction procedures	SI3
Additional comments on Al_2O_3 characterization	SI5
Additional comments on in-flow reactions	SI5
Calculation of the space-time conversion (STC)	SI5
Tables S1-S3	SI7
Figures S1-S16	SI10
Characterization of compounds	SI26
NMR copies	SI29

Experimental Section.

General. Glassware was dried in an oven at 175 ºC before use. Reactions were performed in 6-7 ml vials equipped with a magnetic stirrer and closed with a steel cap having a rubber septum part to sample out. Reagents and solvents were obtained from commercial sources and were used without further purification otherwise indicated. Products were characterised by GC-MS and NMR, and compared with the given literature. The references for the alumina samples used are:

Type of alumina	Commercial source	Reference
Acidic, Brockmann I	Merck	199966
Basic, Brokmann I	Merck	199443
Neutral, Brockmann Activity I	Merck	06300

Physicochemical techniques.

- Inductively coupled plasma-optical emission spectroscopy (ICP-OES): The metal content of the alumina samples was determined after disaggregating the solids in aqua regia, followed by aqueous HF and dilution before analysis. The results were typically 0.1-10 mmol / g of metal cations on the alumina.

- X-ray diffraction (XRD): Spectra were recorded in a CubiX PRO (PAN Analytical) spectrometer, with a Cu K(α) radiation source, 1.5406 Å wavelength.

- Fourier Transformed Infrared (FT-IR) Spectroscopy: Spectra were recorded on attenuated total reflection infrared spectroscopy, from 400 to 4000 cm^{-1}, by dropping a small sample on the ATR crystal.

- Solid state magic angle spinning nuclear magnetic resonance (ss-MAS NMR). Spectra were recorded at room temperature with a Bruker AVIII HD 400 WB spectrometer. The ^{27}Al spectra were recorded with $\pi/12$ pulse length of 1 μs, and a recycle delay of 3s, pinning the samples at 20 kHz.

- Gas chromatography (GC): Gas chromatographic analyses were performed in an instrument equipped with a 25 cm capillary column of 5% phenylmethylsilicone. *N*-

dodecane was used as an external standard. Calibration curves were performed with reactants **1** and **2**, and the isolated products **3** and **4**, after preparing different solutions with varied amounts of each compound, and injecting them in the GC.

- Gas chromatography-mass spectrometry (GC-MS): GC-MS analyses were performed on a spectrometer equipped with the same column as the GC and operated under the same conditions.

- Liquid nuclear magnetic resonance (NMR): ^{1}H, ^{13}C and DEPT NMR spectra were recorded at room temperature on a 300.1 or 400 MHz spectrometer. The range of measurement was from -1 to +12 ppm in ^{1}H-NMR and from 0 to +220 ppm in ^{13}C-NMR.

Typical reaction procedure.

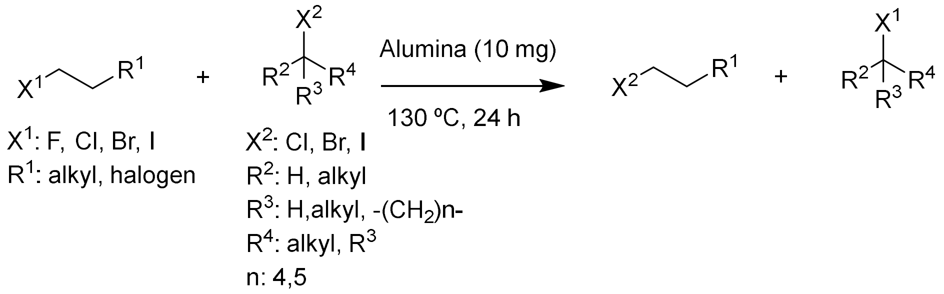

Scheme S1. Halogen exchange reaction conditions.

Products were obtained following the reaction Scheme S1. Reagents (1 mmol and 5-10 eq., 5-10 mmol, respectively) were introduced in 7 ml sealed vials with a magnetic stirrer and then the solid catalyst (10 mg, unless otherwise indicated). The mixture was allowed to react for 1 to 3 days at 130 ºC and atmospheric pressure. GC samples were prepared introducing 25 µL of the reaction mixture in a vial with 1 mL of DCM and *N*-dodecane as an external standard.

Leaching test. Two identical reactions between **1** and **2** were prepared following the procedure described above. For each reaction samples were taken at the same time and, after 60 minutes, one of the reactions was filtered using a PTFE hydrophobic 0.22µm syringe filter and left to react for an additional 23 hours to see if any change in the reactant conversion can be observed.

Reusability test. 1,8-dribromooctane (**1**, 1 mmol) and 1-iodobutane (**2**, 5 equiv., 5 mmol) were introduced in a sealed vial with a magnetic stirrer and then the solid catalyst (10 mg). The mixture was allowed to react for 15 hours at 130 ºC and atmospheric pressure. After reaction time, the catalyst was separated from the solution by centrifugation and cleaned with hexane 5- 6 times. After this treatment, the recovered catalyst was introduced in a vial with a new solution of 1 and 2 and allowed to react another 15 hours. This procedure was repeated 4 times.

D_2O experiments in batch: *Test 1:* α-bromo-γ-butyrolactone **21** (1 mmol) and D_2O (1 equiv., 1 mmol) were introduced in a sealed vial containing a magnetic stirrer and the alumina catalyst (10 mg). *Test 2:* α-bromo-γ-butyrolactone **21** (1 mmol), 1-iodobutane **2** (10 equiv., 10 mmol) and D_2O (1 equiv., 1 mmol) were introduced in a sealed vial containing a magnetic stirrer and the alumina catalyst (10 mg). The mixtures was allowed to react for 24 hours in a pre-heated oil bath at 130 ºC under atmospheric pressure. Aliquots (typically 25 μl) were periodically taken from the supernatant, and gas chromatography (GC) samples were prepared after dilution of the reaction mixture in a vial with 1 mL of dichloromethane (DCM) and *n*-decane as an external standard. Products were characterized by gas chromatography-mass spectrometry (GC-MS).

In flow reaction procedure. α-bromo-γ-butyrolactone **21** (35 mmol) and 1-iodobutane **2** (10 equiv., 350 mmol) were placed in a 50.0 mL syringe. The mixture was pumped in counter-gravity mode at atmospheric pressure and at a flow of 0.1 $mL\cdot min^{-1}$, on the bottom of a stainless-steel tube with a 1-inch internal diameter and filled with 10 g of chromatographic grade pelletized Na^+-Al_2O_3 (sieved to a particle size of 0.4-0.8 mm). The reaction took place at 130 ºC and samples were collected by gravity after passing through an U-tube. The samples were analysed by gas chromatography (GC) after dilution of the reaction mixture in a vial with 1 mL of DCM and *n*-decane as an external standard.

For the experiment with D_2O, we added 1 equivalent to the mixture in the syringe.

Coupling reactions (see Figure 5).

A) To a 0.1 mmol of diiodoctane **4** diluted in 1 mL of acetonitrile (0.1M), we added 0.11 mmol (23.0 mg) of *N,N′*-dicyclohexyl- 1,2-ethanediamine and 0.11 mmol of K_2CO_3 (15.2 mg), heating at 90 ºC for 24 h. After that, the reaction mixture was

cooled down to room temperature, quenched with water and extracted with ethylacetate (3 × 2.0 mL). The combined organic layers were washed with brine (5.0 mL), dried with anhydrous $MgSO_4$, and concentrated in vacuo.

B) To a 0.1 mmol of dibromooctane **1** or diiodoctane **4** diluted in 1 mL of acetonitrile (0.1M), we added 0.22 mmol of *N*-ethylaniline (25.0 mg) and 0.11 mmol of K_2CO_3 (15.2 mg), heating at 90 ºC for 24h. After that, the reaction mixture was cooled down to room temperature, quenched with water and extracted with ethylacetate (3 × 2.0 mL). The combined organic layers were washed with brine (5.0 mL), dried with anhydrous $MgSO_4$, and concentrated in vacuo.

Additional comments on Al_2O_3 characterization

The XRD diffractograms indicate a typical γ phase for alumina, with two different aluminium coordination environments: Al^{IV} (5 ppm) and Al^{VI} (60 ppm), but without Al^{V} (35 ppm), by ^{27}Al ss-MAS NMR. The intensity of the penta-coordinated peak relates to the disorder in the alumina. BET surface measurements show that the alumina is mesoporous, which allows a good molecular traffic. In accordance with the XRD analysis, the ^{27}Al ss-MAS NMR spectra show that most of Al oxide is in tetra-coordinated form (>75%, bulk oxide) and the rest in hexa-coordinated form (<25%, surface oxide).

Additional comments on in-flow reactions.

In order to further increase the efficiency per time of the alumina catalyst, a new experiment in flow was carried out, with 30 times less alumina (0.35 g) in the tubular reactor. The results (Figure S13a) show that the conversion decreases from 100% to ~70%, and also the selectivity to product **4**. However, both values could be significantly increased after decreasing the reaction flow rate to 0.03 ml/min, to achieve a STC of 0.31 mol of **1** / (L·h), higher than with 10 g of alumina (see also Figures 3 and S7).

Calculation of the space-time conversion (STC)

Calculation of the space-time conversion (STC) for the tubular reactor and comparison with the batch reactor.

<u>Flow reactor:</u> 10 g of solid catalyst, 0.1 $mL \cdot min^{-1}$ (flow rate, 1600 min reaction time, 13.9 % *v:v* of limiting reactant **1** in the reaction mixture, >99% conversion, thus:

$$0.1\ mL \cdot min^{-1} \cdot 1600\ min \cdot 0.139\ (ml\ \mathbf{1}\ /\ ml\ mixture):\ 22.2\ ml\ \mathbf{1}\ converted.$$

22.2 ml **1** correspond to 121 mmol. Catalyst volume is 19.4 mL (10 g) and time is 26 h:

$$Space - time\ conversion\ (STC) = \frac{121 \cdot 10^{-3}\ mol}{19.4 \cdot 10^{-3} L \cdot 26\ h} = 0.24 \frac{mol}{L \cdot h}$$

<u>Batch reactor:</u> 10 mg of solid catalyst, 24 h reaction time.

1 mmol of limiting reactant with a 99.0 % of conversion gives 0.99 mmol of product. Reaction volume is 2.0 mL and time is 24 h.

$$STC = \frac{0.99 \cdot 10^{-3}\ mol}{2 \cdot 10^{-3} L \cdot 24\ h} = 0.021 \frac{mol}{L \cdot h}$$

Tables.

Table S1. Catalytic results for the halex reaction between dibromooctane **1** and iodobutane **2** with different alumina samples. Reaction conditions: 1,8-dibromooctane **1** (1 mmol), 1-iodobutane **2** (10 mmol), 10 mg of alumina (3.5 wt% respect to **1**), ambient atmosphere, 130 ºC, 24 h. Conversion refers to the total conversion of **1** to the mixture of products **3** + **4**. [a] The amount of the corresponding countercations was obtained by ICP-OES, except for Cs^{+} that is not possible to analyze it.

Entry	Countercation	Amount of counteraction (mmol/g)[a]	Conv. (%)	Select. **3** (%)	Select. **4** (%)	Initial rate (conv. %/ h)
1	Ca^{2+}	0.13	99	17	82	50.4
2	Cs^{+}	--	81	61	39	37.5
3	K^{+}	10.1	91	45	55	45.4
4	Na^{+}	0.12	99	17	83	75.6
5	Li^{+}	4.96	69	70	30	40.1
6	H^{+}	--	99	7	93	30.0

Table S2. Brunauer-Emmett-Teller (BET) surface area results for Na^{+}-Al_2O_3 before and after pelletizing.

Na-Al_2O_3	Area		
	BET surface area	Micropore area	Micropore volume (cm3/g)
Powder	155.6359 m^2/g	0 m^2/g (negative micropore volume)	-0.070515 cm^3/g
Pellets	191.9959 m^2/g	0 m^2/g (negative micropore volume)	-0.064567 cm^3/g

Table S3. ^{27}Al solid state magic angle spinning nuclear magnetic resonance (^{27}Al ss-MAS NMR) integration results for Na^+-Al_2O_3 before and after pelletizing. Tetrahedral Al (5 ppm), pentahedral Al (35 ppm) and hexahedral Al (60 ppm).

^{27}Al NMR			
Na-Al_2O_3	Integration area, 60 ppm	Integration area, 35 ppm	Integration area, 5 ppm
Powder	0.232	0	0.775
Pellets	0.242	0	0.758
Pellets after reaction	0.211	0	0.789

Figures.

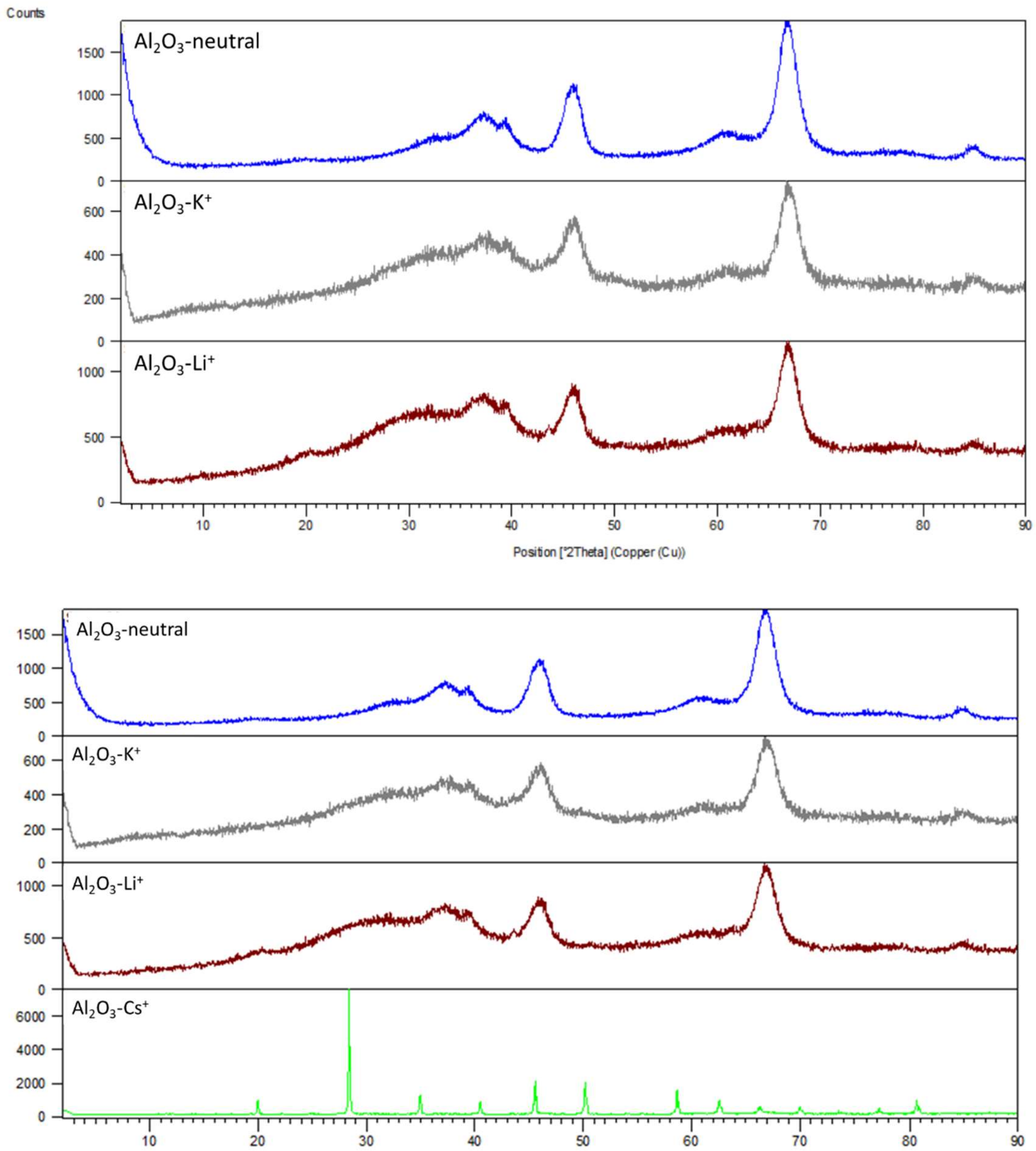

Figure S1. X-ray diffraction (XRD) measurements of neutral-Al_2O_3 and after cation exchange with K^+, Li^+ and Cs^+.

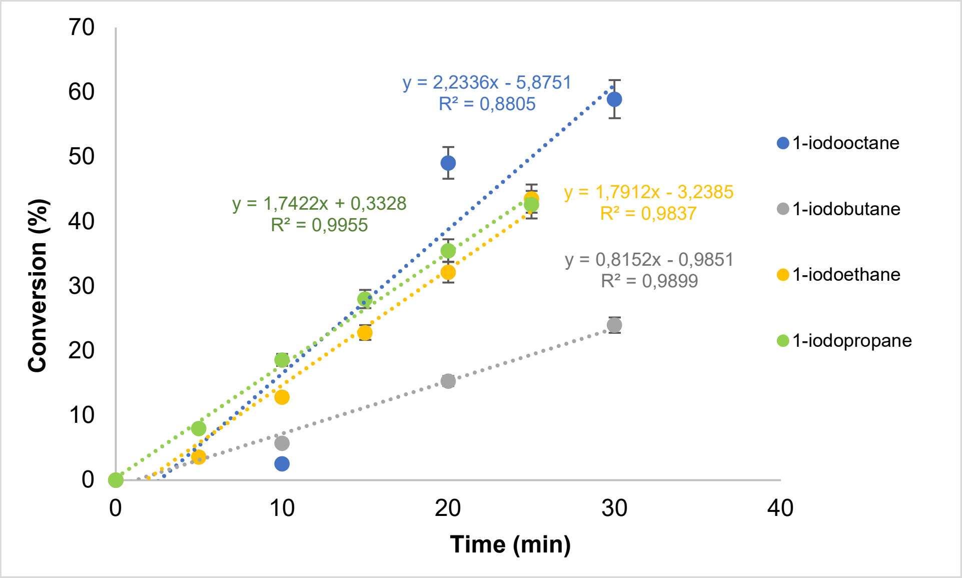

Alkyl chain	Initial rate (conv. % / h)
C2	1.8
C3	1.7
C4	0.8
C8	2.2

Figure S2. Initial rate depending on the length or structure of the molecule. Reaction conditions in Table 1 of the manuscript. Error bars account for a 5% uncertainty.

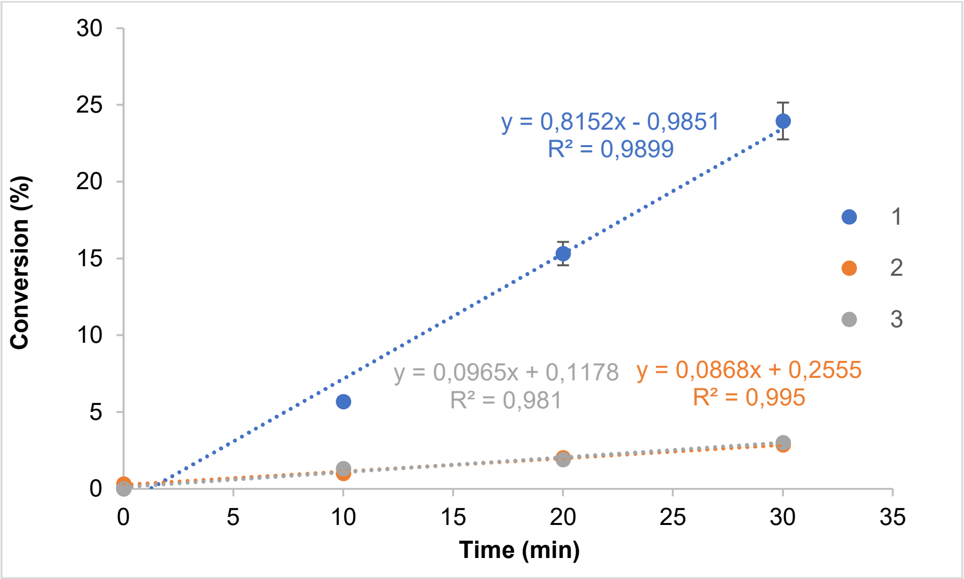

Figure S3. Initial rate depending on the limitant reactant. Reaction conditions in Table 1 of the manuscript. 1: Iodobutane **2** (5. Equiv.) + dibromooctane **1** (1 mmol). 2: Iodobutane **2** (5 mmol) + dibromooctane **1** (1 equiv.). 3: Iodobutane **2** (1 mmol) + dibromooctane **1** (5 equiv.). Error bars account for a 5% uncertainty.

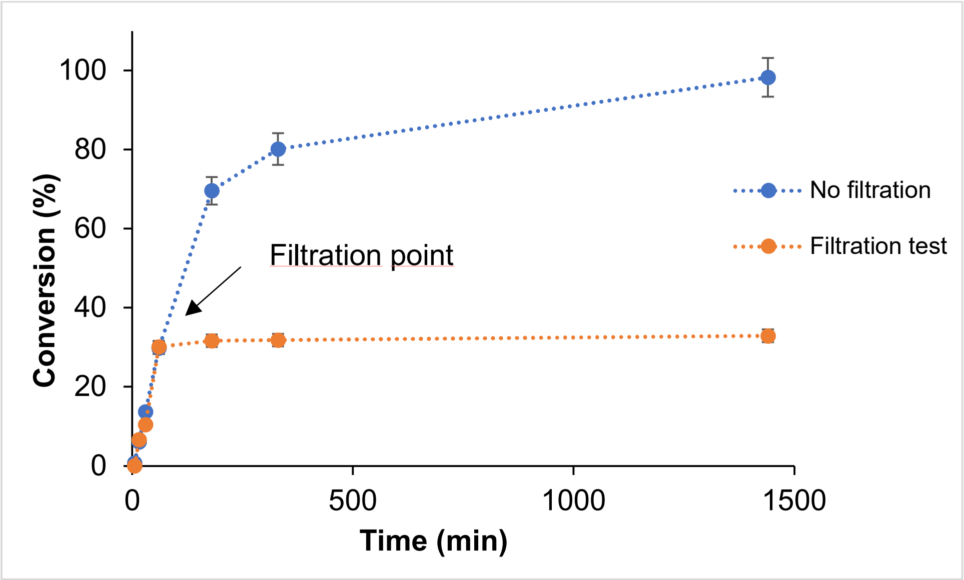

Figure S4. Leaching test for the halex reaction catalyzed by Na^{+}-Al_2O_3, for reaction conditions see Table 1 in the main text. Error bars account for a 5% uncertainty.

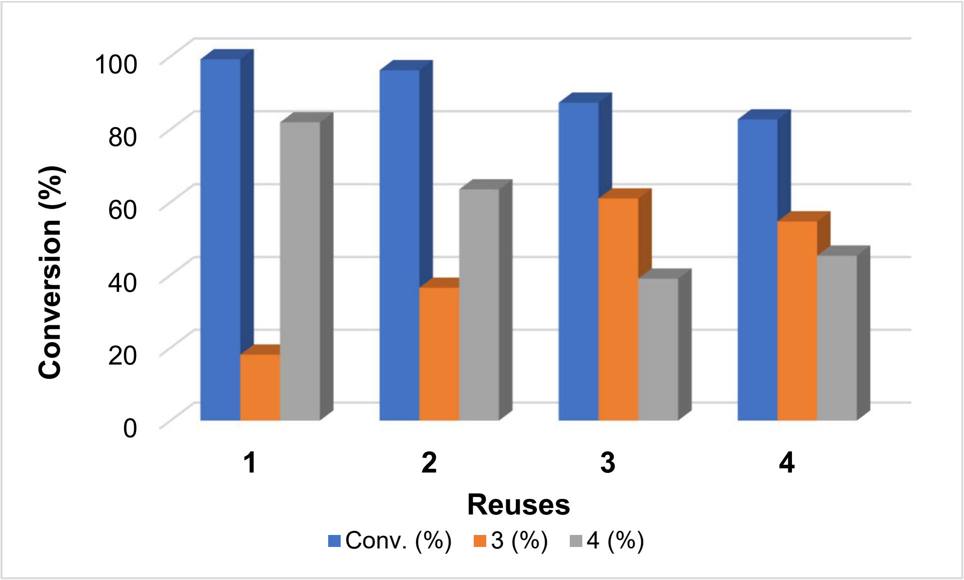

Figure S5. Reuses of Na^+-Al_2O_3 catalyst, in batch, for the halex reaction, after 15h reaction time. For the rest of reaction conditions, see Table 1 in the main text.

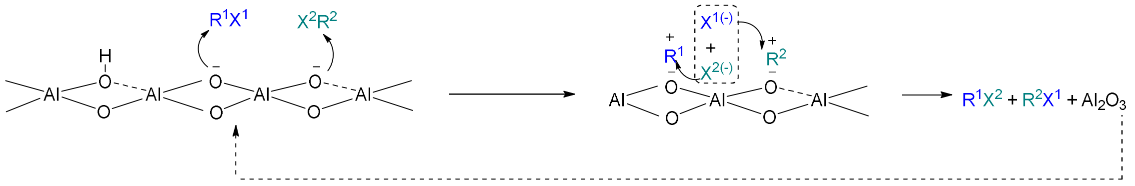

Figure S6. Reaction mechanism proposed for the halex reaction with alumina as a catalyst.

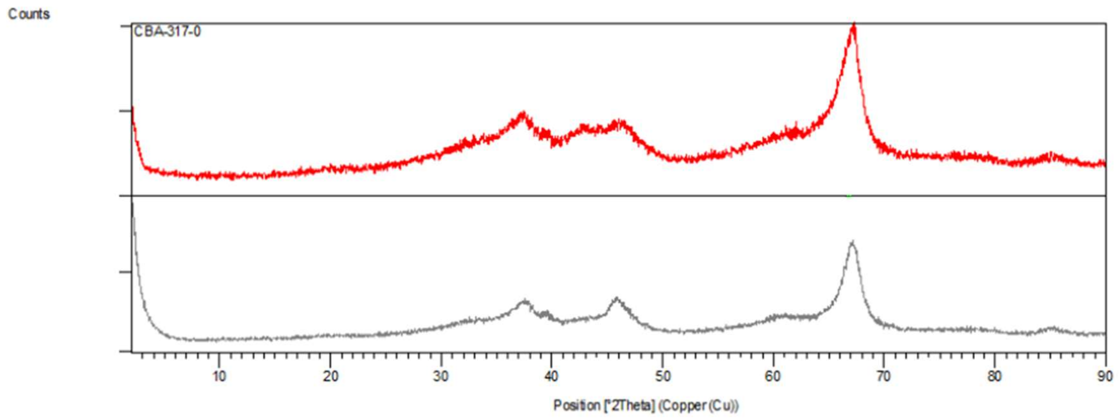

Figure S7. X-ray diffraction (XRD) measurements of Na^+-Al_2O_3 before (bottom, grey) and after pelletizing (top, red).

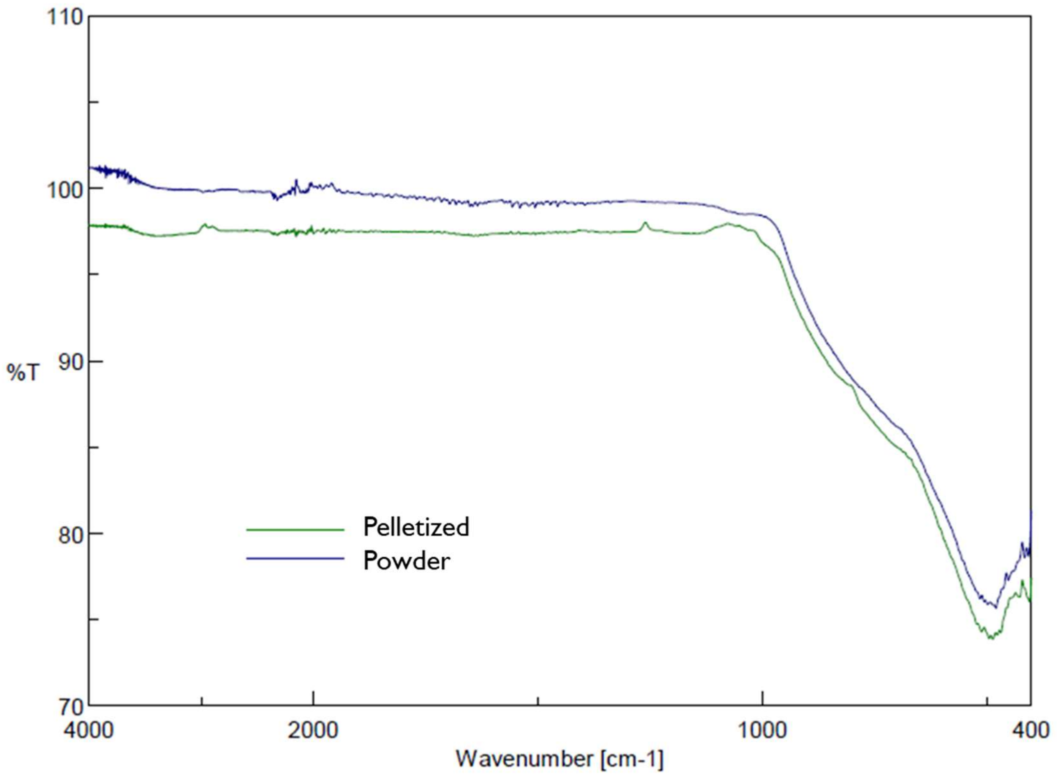

Figure S8. Fourier transform infrared (FT-IR) spectra of Na^{+}-Al_2O_3 before (blue line) and after pelletizing (green line).

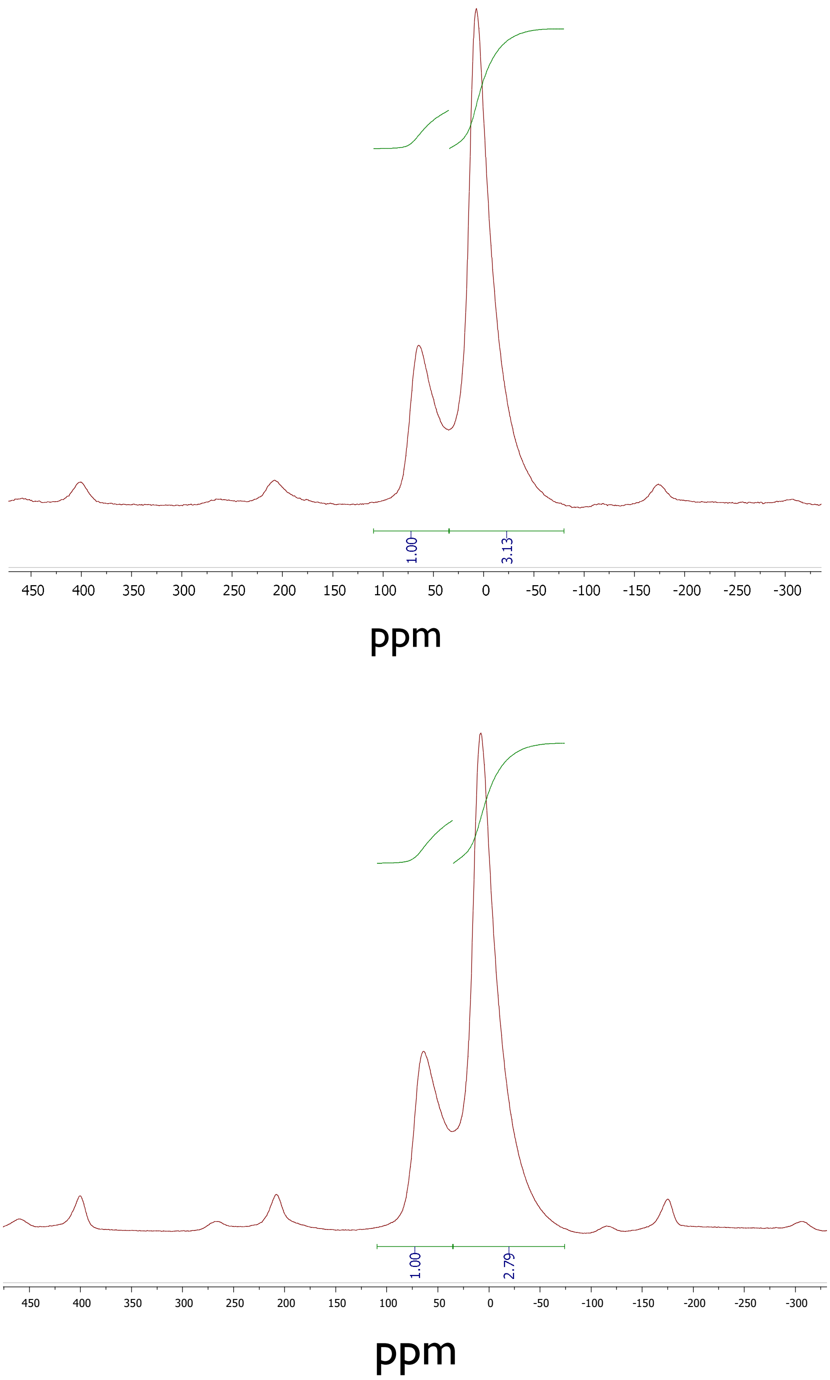

Figure S9 ^{27}Al solid state magic angle spinning nuclear magnetic resonance (^{27}Al ss-MAS NMR) spectra of Na^{+}-Al_2O_3 before (bottom) and after pelletizing (top, see also Table S3).

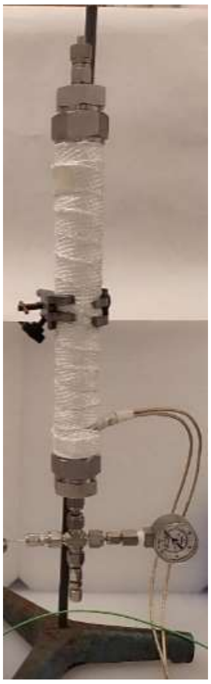

Figure S10. A photograph of the in-house made tubular reactor employed in this study.

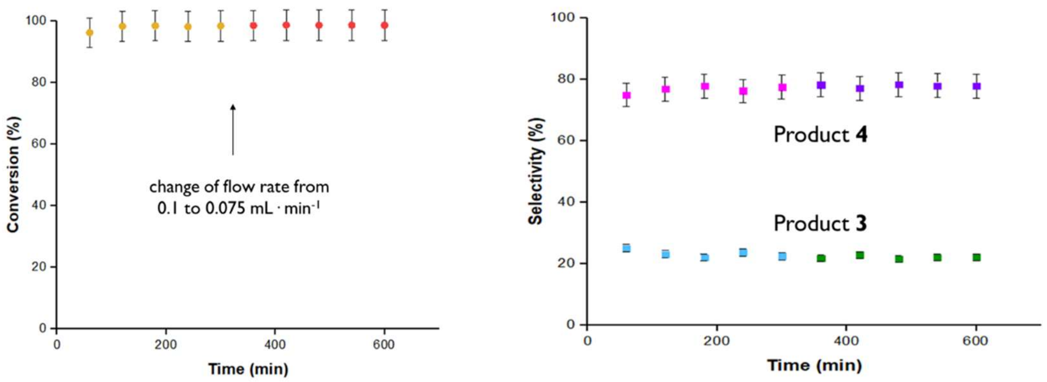

Figure S11. Conversion (left) and selectivity (right) results for the in-flow halex reaction of **1** and **2** with 10.7 g of Na^+-Al_2O_3 catalyst, pelletized between 0.4-0.8 μm, and placed in a fixed-bed tubular (1 cm diameter, 30 cm length) with a reactant feed flow rate of 0.1 mL·min^{-1} (first 350 min) and then 0.075 mL·min^{-1}, at 130 °C. Error bars account for 5% uncertainty.

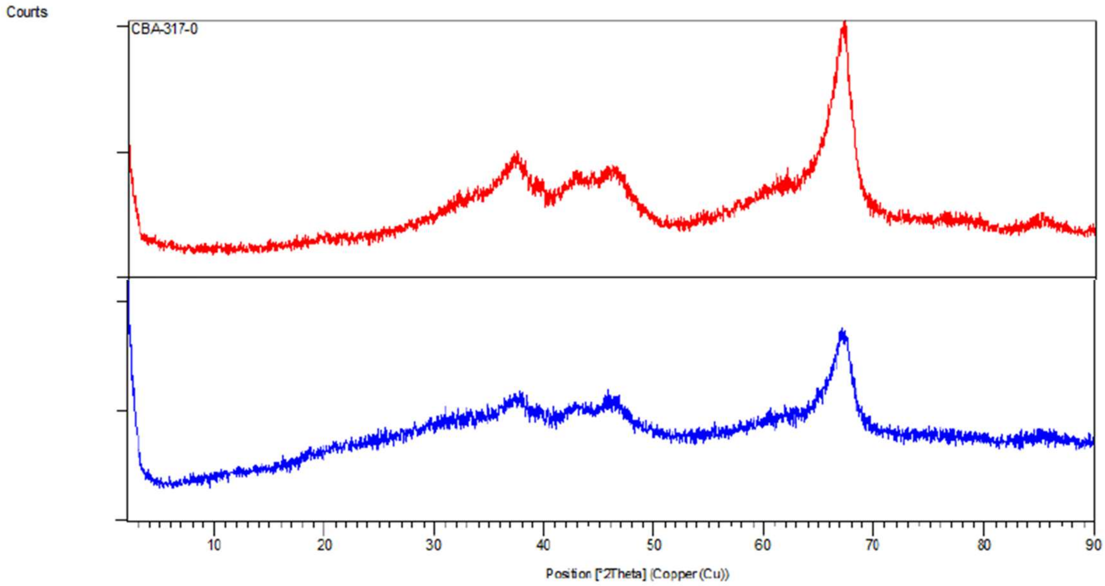

Figure S12. X-ray diffraction (XRD) measurements of Na^{+}-Al_2O_3 before (top, red) and after reaction in the tubular reactor for 26 h reaction time (bottom, blue).

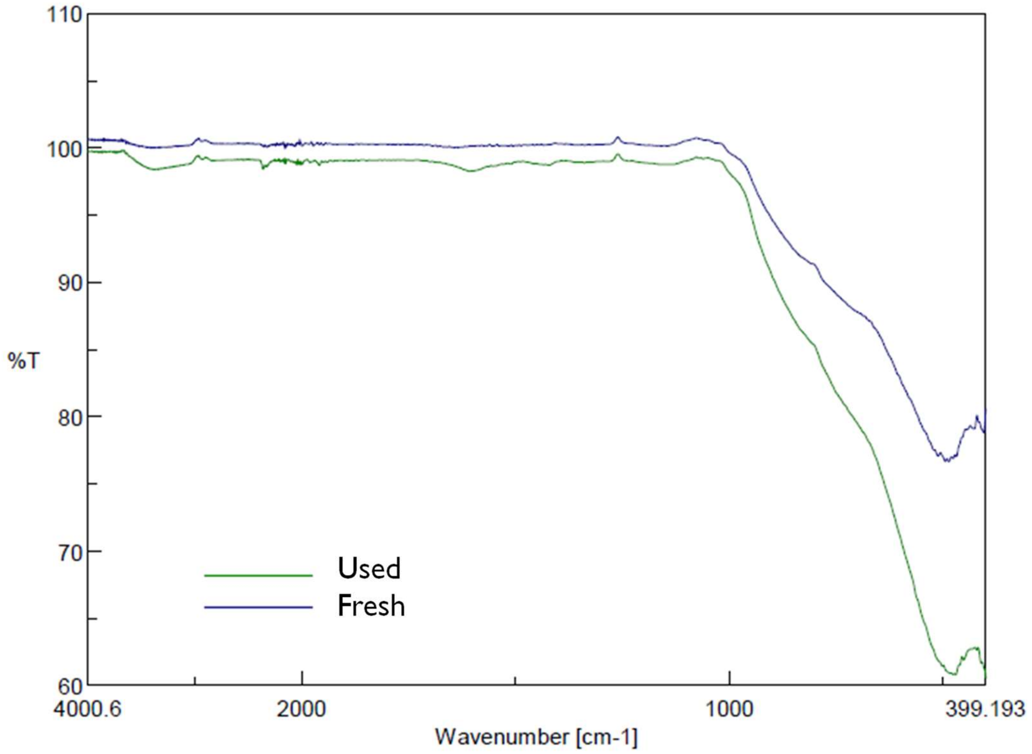

Figure S13. Fourier transform infrared (FT-IR) spectra of Na^{+}-Al_2O_3 before (blue line) and after reaction in the tubular reactor for 26 h reaction time (green line).

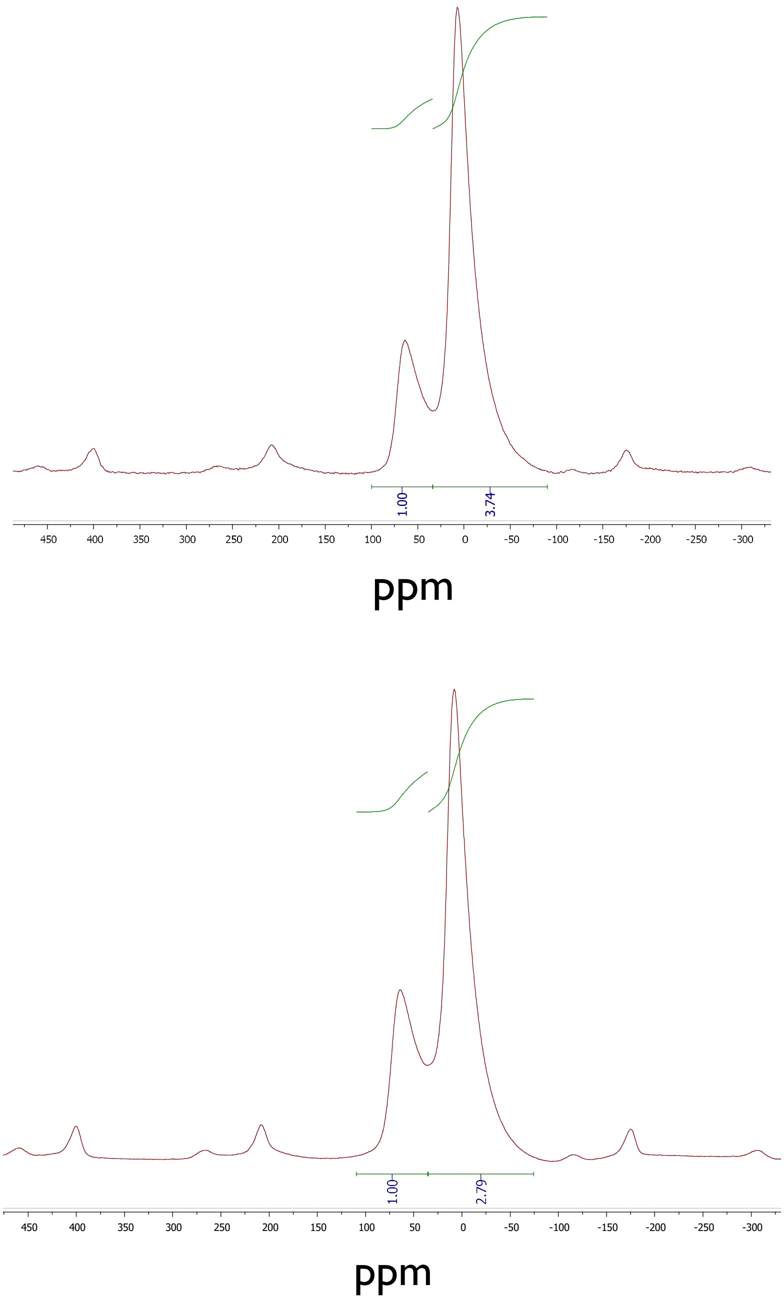

Figure S14. ^{27}Al solid state magic angle spinning nuclear magnetic resonance (^{27}Al ss-MAS NMR) spectra of Na^+-Al_2O_3 before (bottom) and after reaction in the tubular reactor for 26 h reaction time (top, see also Table S3).

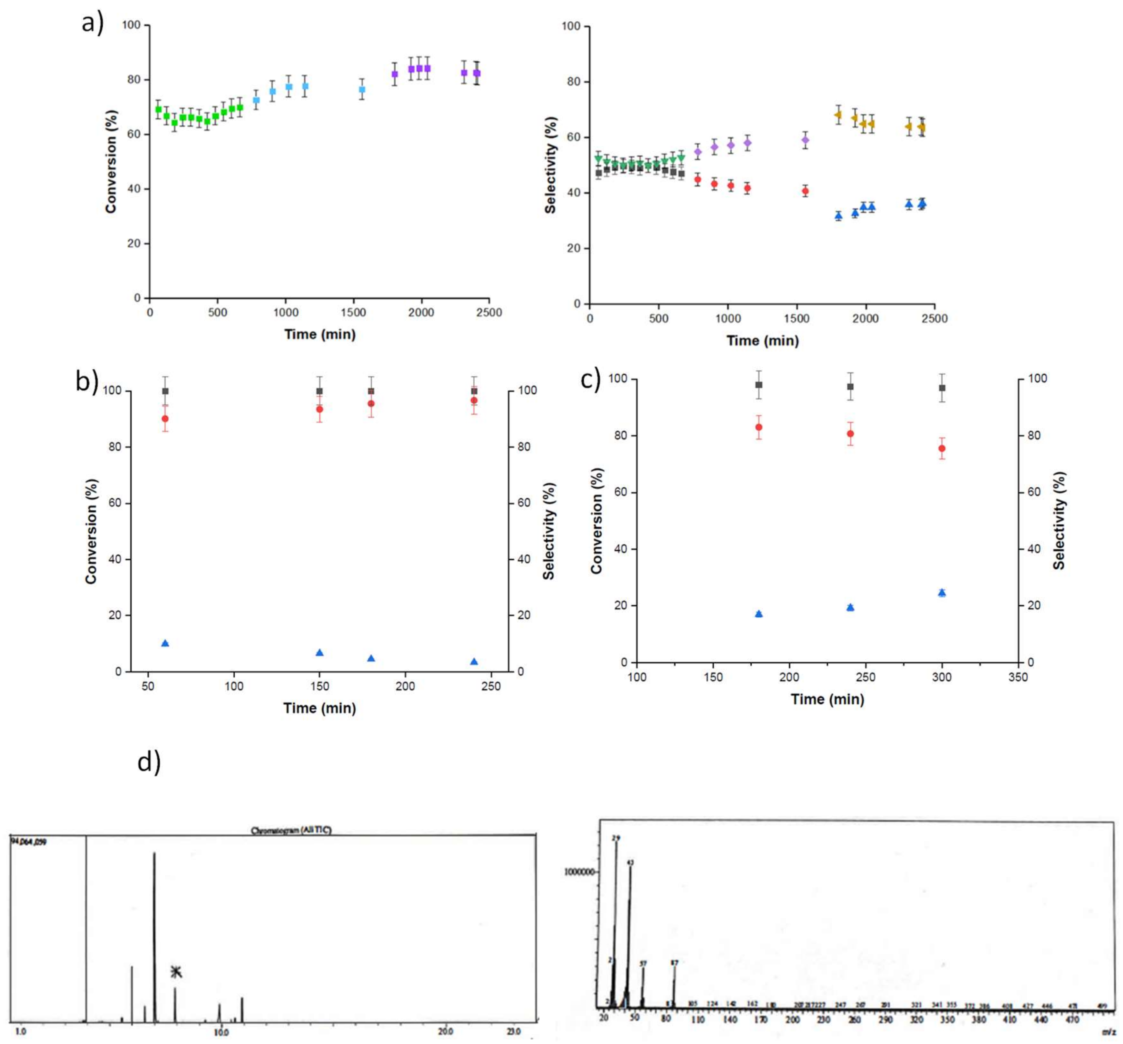

Figure S15. a) Conversion (left) and selectivity (right) results for the in-flow halex reaction of **1** and **2** with 350 mg of Na^+-Al_2O_3 catalyst, pelletized between 0.4-0.8 μm, placed in a fixed-bed tubular (1 inch diameter, 30 cm length) with a reactant feed flow rate of 0.1 mL·min^{-1} (green points, up to 800 min reaction time), 0.05 mL·min^{-1} (light blue points, up to 1600 min reaction time) and 0.03 mL·min^{-1} (purple points, up to 2400 min reaction time), at 130 ºC. The selectivity is always higher for **4** (green, purple and yellow points) than for **3** (black, red and blue points on the left graph). b) Conversion results for the in-flow halex reaction of **21** and **2** with 10 g of Na^+-Al_2O_3 catalyst, pelletized as above and with a reactant feed flow rate of 0.1 mL·min^{-1} (black points, up to 200 min reaction time), at 130 ºC. The selectivity is always higher for **34** (red points) than for **22** (blue points). c) Conversion results for the in-flow halex reaction of **21** and **2** in presence of D_2O (reaction conditions as above, black points, up to 300 min reaction time at 130 ºC. The selectivity is always higher for **34-*d*$_1$** (red points) than for **22** (blue points). d) GC-MS of the **34-*d*$_1$** product obtained. Error bars account for 5% uncertainty.

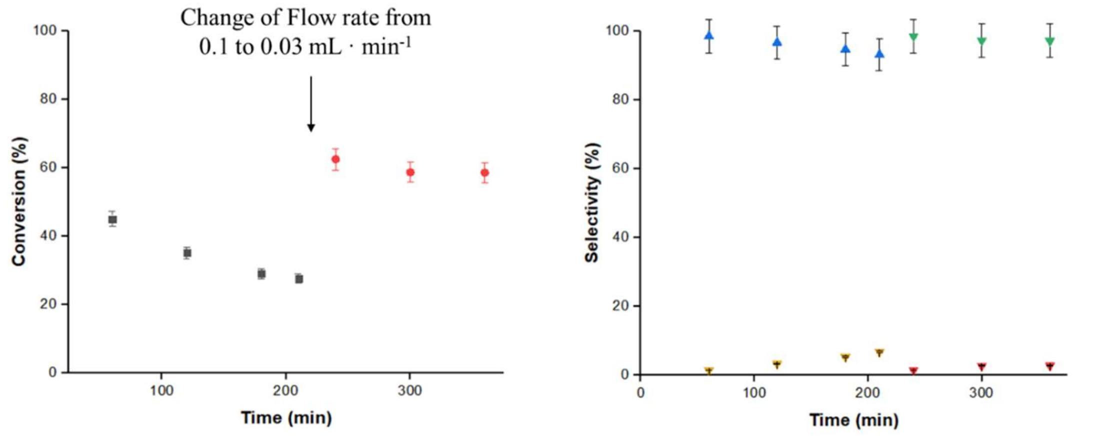

Figure S16. Conversion of 1-fluorooctane **9** (black squares up to 200 min reaction time, red points up to 360 min reaction time), and selectivity towards 1-iodooctane **8** (yellow triangles up to 200 min reaction time, red triangles up to 360 min reaction time) or 1-octene **35** (blue triangles up to 200 min reaction time, green triangles up to 360 min reaction time), during the in-flow halex reaction of **9** and **2** with 350 mg of Na^{+}-Al_2O_3 catalyst and different flow rates, at 130 ºC. Ether products were not found. Error bars account for 5% uncertainty.

Characterization of compounds.

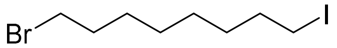
1-bromo-8-iodooctane **(3).** Orange liquid, 358.5 mg of mixture **3** and **4** (25.5:72.5), 98.0% isolated yield. GC-MS (m/z, $M^{+\cdot}$319), major peaks found: 320-318, 193-191 (-I), 111 (100%, -Br), 69, 55, 41, 27. ^{1}H NMR (401 MHz, $CDCl_3$): δ 3.39 (t, *J* = 6.8 Hz, 2H), 3.19 (t, *J* = 6.8 Hz, 2H), 1.81 (m, 4H), 1.42 – 1.29 (m, 8H). ^{13}C{1H} NMR (101 MHz, $CDCl_3$): δ 33.9 (CH_2Br), 33.4 (CH_2), 32.7 (CH_2), 30.3 (CH_2), 28.5 (CH_2), 28.2 (CH_2), 28.0 (CH_2), 7.2 (CH_2I).

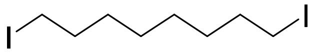
1,8-diiodooctane **(4).** Orange liquid, 358.5 mg of mixture **3** and **4** (25.5:72.5), 98.0% isolated yield. GC-MS (m/z, $M^{+\cdot}$ 366), major peaks found: 366, 239 (-I), 183, 155, 111 (-I), 69 (100%), 55, 41. ^{1}H NMR (401 MHz, $CDCl_3$) δ 3.19 (t, *J* = 6.8 Hz, 4H), 1.81 (m, 4H), 1.42 – 1.29 (m, 8H). ^{13}C{1H} NMR (101 MHz, $CDCl_3$): δ 33.4 (CH_2), 30.3 (CH_2), 28.2 (CH_2), 7.2(CH_2I).

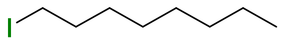
1-iodooctane **(8).** GC-MS (m/z, M^{+} 240), major peaks found: 240 (100%), 155, 113 (-I), 71, 57, 43, 29.

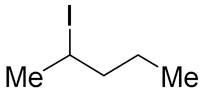
2-iodopentane **(11).** GC-MS (m/z, $M^{+\cdot}$198), major peaks found: 198, 127 (I), 71 (100%, -I), 55, 43, 29.

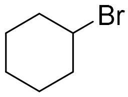
Bromocyclohexane **(14).** GC-MS (m/z, $M^{+\cdot}$163), major peaks found: 164-162, 83 (100%, -Br), 67, 55, 41, 27.

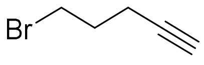
5-bromo-1-pentyne **(18).** GC-MS (m/z, $M^{+\cdot}$147), major peaks found: 148-146, 67 (100%, -Br), 39.

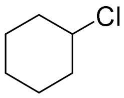
Chlorocyclohexane **(15).** GC-MS (m/z, $M^{+\cdot}$ 119), major peaks found: 120-118, 82 (100%), -Cl), 67, 55, 41, 28.

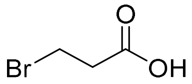
3-bromopropanoic acid **(20).** GC-MS (m/z, $M^{+\cdot}$153), major peaks found: 154-152, 137-135, 109-107, 73 (-Br), 55, 43 (100%), 27.

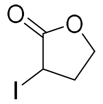 *α-iodo-γ-butyrolactone* **(22).** GC-MS (m/z, $M^{+\cdot}$212), major peaks found: 213 (100%), 86 (-I), 69, 57, 41.

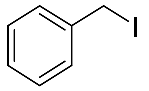 *(Iodomethyl)benzene* **(22)**. GC-MS (m/z, $M^{+\cdot}$218), major peaks found: 218, 127 (I), 91 (100%, -I), 65.

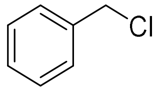 *(Chloromethyl)benzene* **(24)**. 20.3% isolated yield, brown liquid. GC-MS (m/z, $M^{+\cdot}$126), major peaks found: 128-126, 91 (100%), 65. ^{1}H NMR (401 MHz, $CDCl_3$): δ 7.52-6.97 (m, 5H), 4.56 (d, J = 6.0 Hz, 2H). ^{13}C NMR (101 MHz, $CDCl_3$): δ 135.5 (C), 129.3 (2CH), 127.8 (2CH), 127.0 (CH), 41.6 (CH_2Cl).

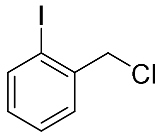 *1-(chloromethyl)-2-iodobenzene* (**28**). GC-MS (m/z, $M^{+\cdot}$253), major peaks found: 254-252, 217 (100%, -Cl), 127 (I), 89 (-I), 63.

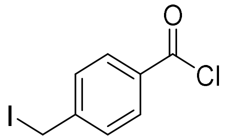 *4-(iodomethyl)benzoyl chloride* (**30**), 68.7% isolated yield, brown solid. GC-MS (m/z, $M^{+\cdot}$280), major peaks found: 282-280, 245 (-Cl), 155-153 (100%, -I), 118, 105, 89. ^{1}H NMR (401 MHz, $CDCl_3$) δ 8.05 (d, J = 8.4 Hz, 2H), 7.50 (d, J = 8.4 Hz, 2H), 4.47 (s, 1H). ^{13}C{1H} NMR (101 MHz, $CDCl_3$) δ 167.7 (C), 147.3 (C), 132.5 (C), 131.9 (CH), 129.3 (CH), 2.6 (CH_2I).

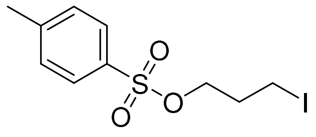 *1-((3-iodopropyl)sulfonyl)-4-methylbenzene* (**32**). GC-MS (m/z, $M^{+\cdot}$ 340), major peaks found: 340, 213 (100%, -I), 172 (-TsO), 155, 91, 65.

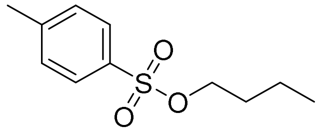 *4-methylbenzenesulfonate* (**33**). GC-MS (m/z, $M^{+\cdot}$ 228), major peaks found: 228, 173 (100%, -TsO), 155, 91, 56.

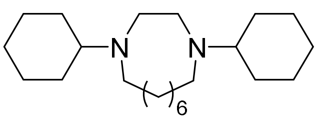 1,4-dicyclohexyl-1,4-diazacyclododecane (**37**). Orange solid, 27.7 mg starting from 0.1 mmol, 81.1% isolated yield. GC-MS (m/z, $M^{+\cdot}$ 334), major peaks found: 334, 251 (100%), 222, 209, 180, 152, 126, 110, 82, 55. ^{1}H NMR (401 MHz, $CDCl_3$) δ 2.76 (s, 4H), 2.64 (m, 2H), 2.43 (m, 4H), 1.77 (m, 8H), 1.20 (m, 24H). ^{13}C{1H} NMR (75 MHz, $CDCl_3$) δ 56.9 (CH), 50.6 (CH_2), 46.4 (CH_2), 33.4 (CH_2), 29.1 (CH_2), 26.3 (CH_2), 26.2 (CH_2), 26.0 (CH_2), 25.0 (CH_2).

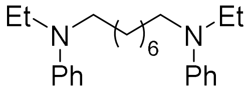 *N^{1},N^{8}*-diethyl-*N^{1},N^{8}*-diphenyloctane-1,8-diamine (**39**). Orange solid, 28.2 mg starting from 0.1 mmol, 80.1 % isolated yield. GC-MS (m/z, $M^{+\cdot}$ 352), major peaks found: 352, 323, 134 (100%), 106, 77. ^{1}H NMR (300 MHz, $CDCl_3$) δ 7.23 (t, *J* = 8.7 Hz, 4H), 6.70 (m, 6H), 3.37 (q, *J* = 7.2 Hz, 4H), 3.25 (t, *J* = 7.8 Hz, 4H), 1.59 (m, 4H), 1.34 (m, 8H), 1.16 (t, *J* = 7.1 Hz, 6H). ^{13}C{1H} NMR (101 MHz, $CDCl_3$) δ 148. (2C), 129.2 (4CH), 115.2 (2CH), 111.8 (4CH), 50.4 ($2CH_2$), 44.9 (CH_2), 29.6 (CH_2), 27.5 (CH_2), 27.2 (CH_2), 12.3 (CH_3).

NMR copies.

1,8-diiodooctane (**4**) with minor amount of **3**.

^{1}H NMR 400 MHz

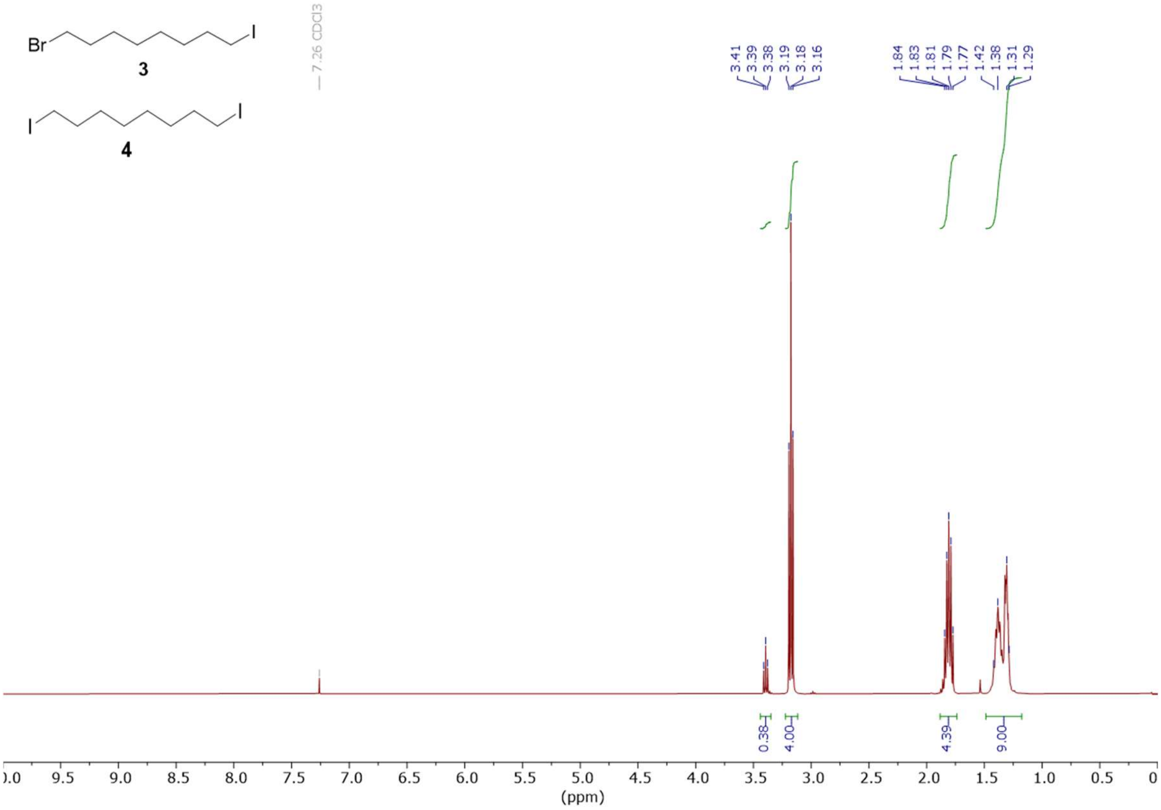

^{13}C NMR 101 MHz

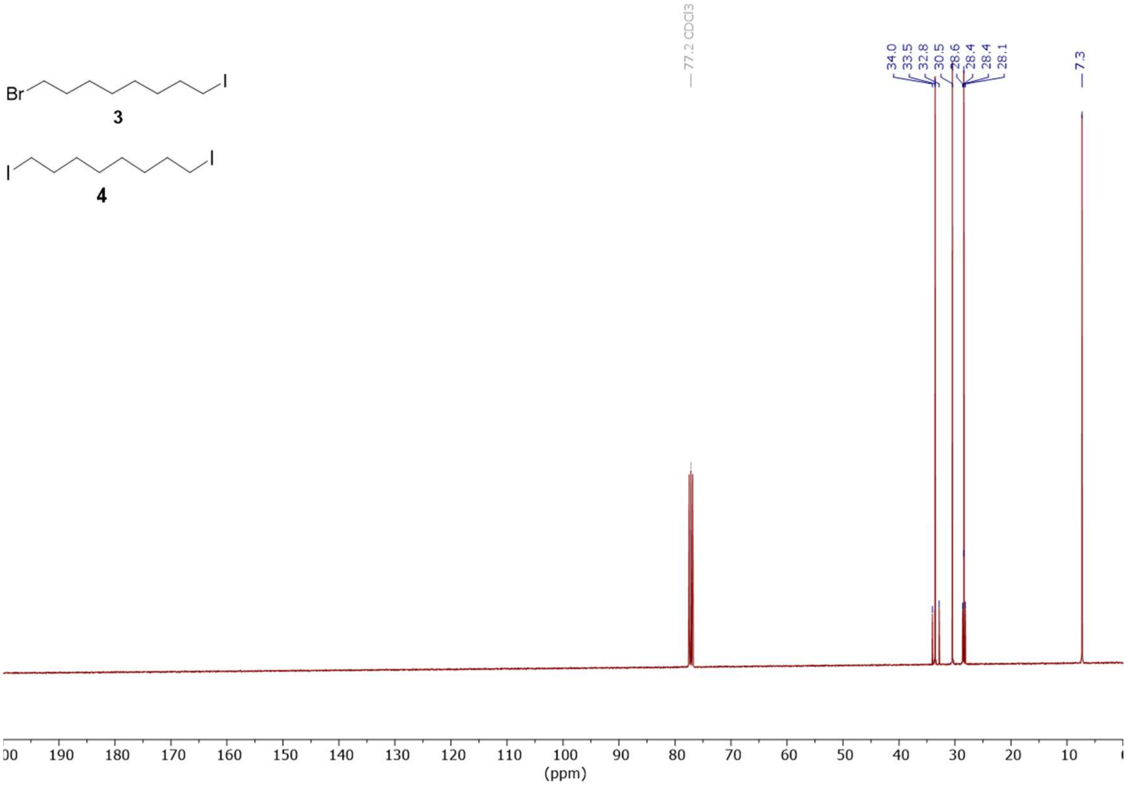

DEPT

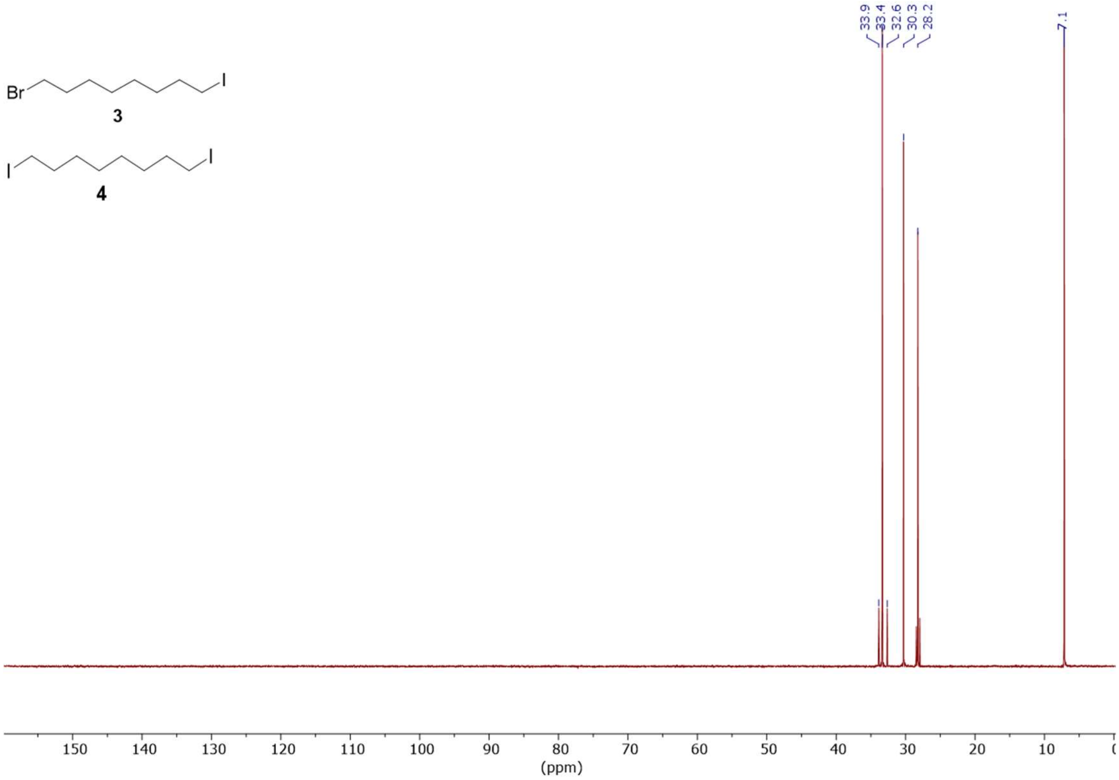

4-(iodomethyl)benzoyl chloride (**30**) with minor amount of **2** and **29**.

^{1}H NMR 400 MHz

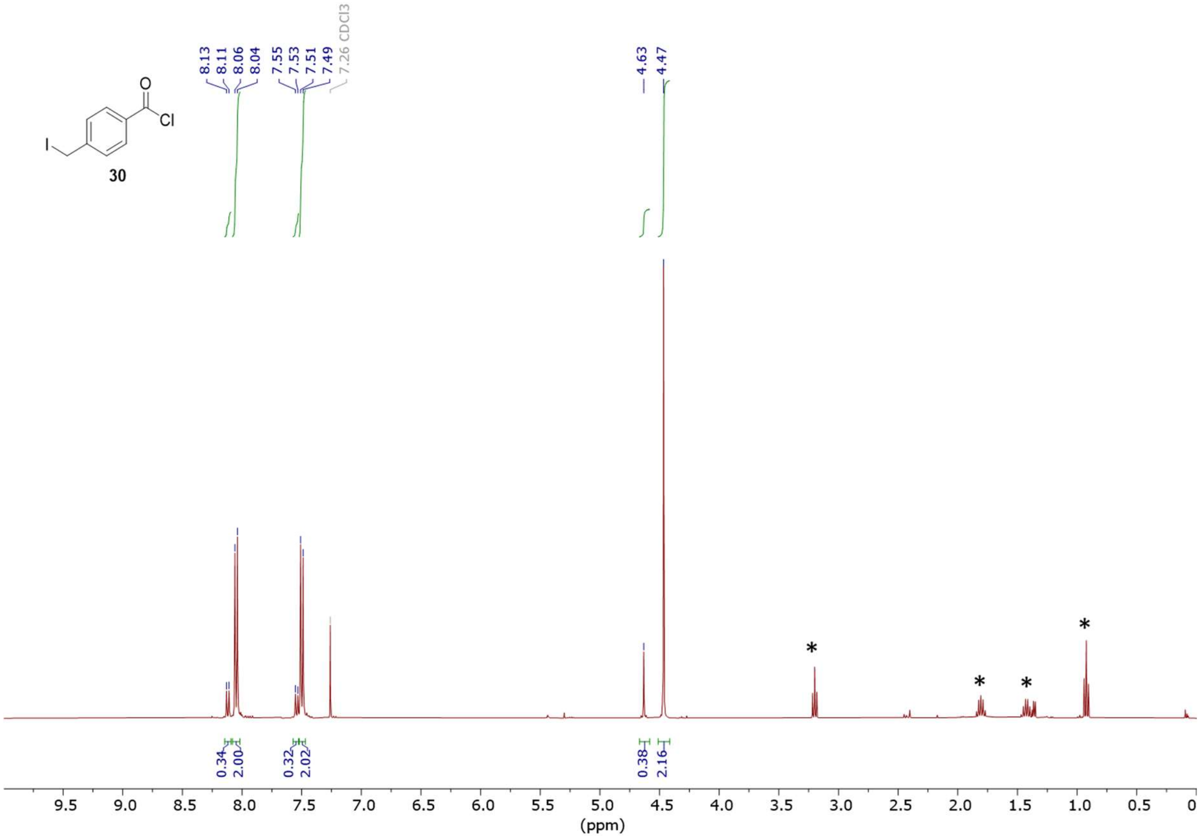

^{13}C NMR 101 MHz

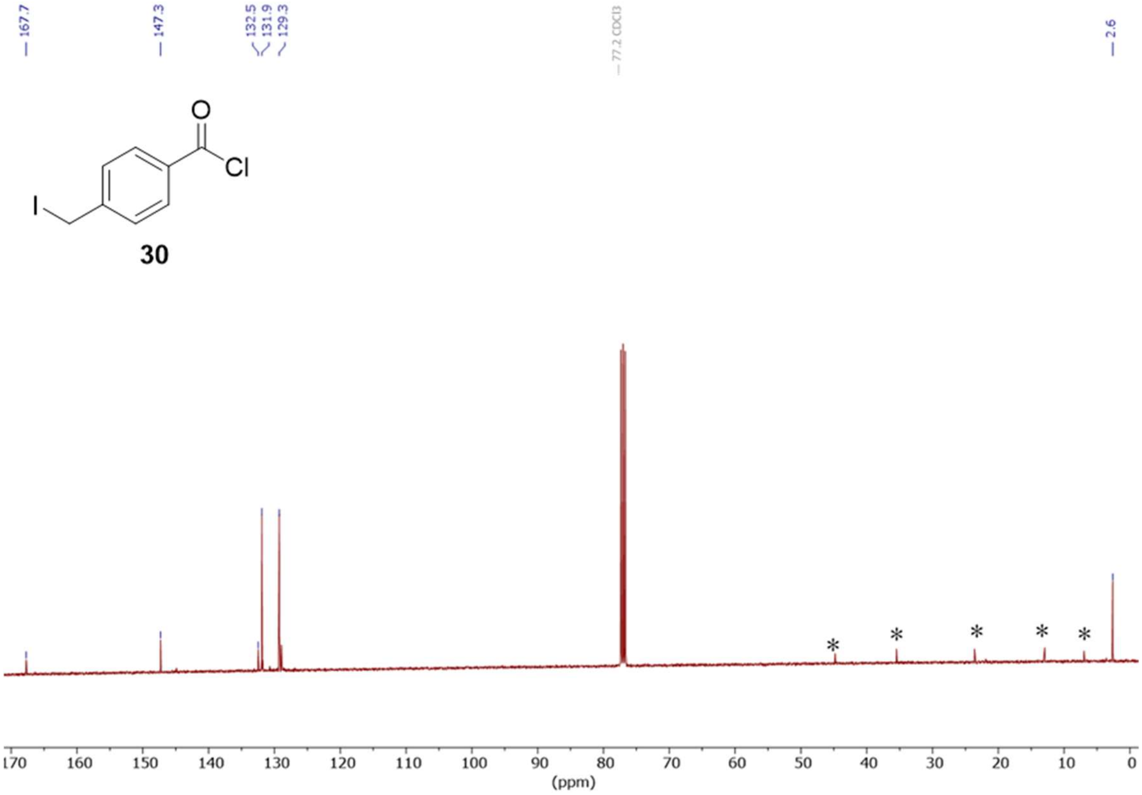

DEPT

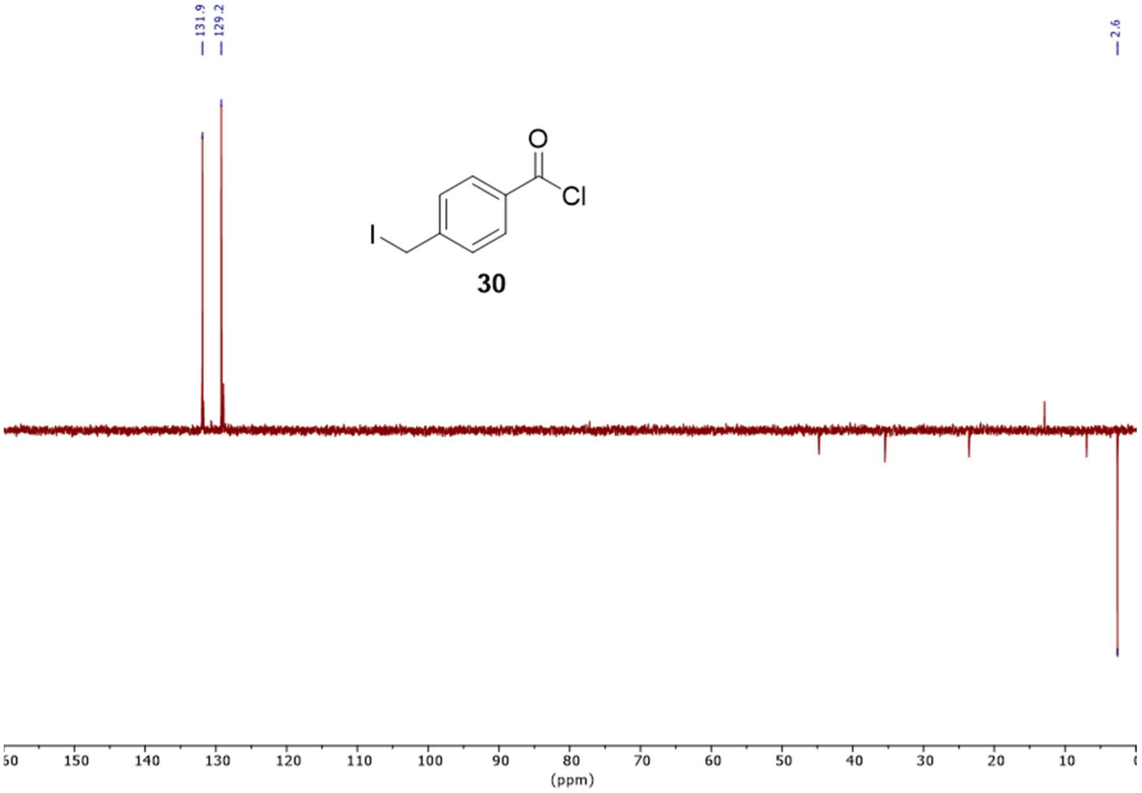

N^{1},N^{8}-diethyl-N^{1},N^{8}-diphenyloctane-1,8-diamine (**39**).

^{1}H NMR 300 MHz

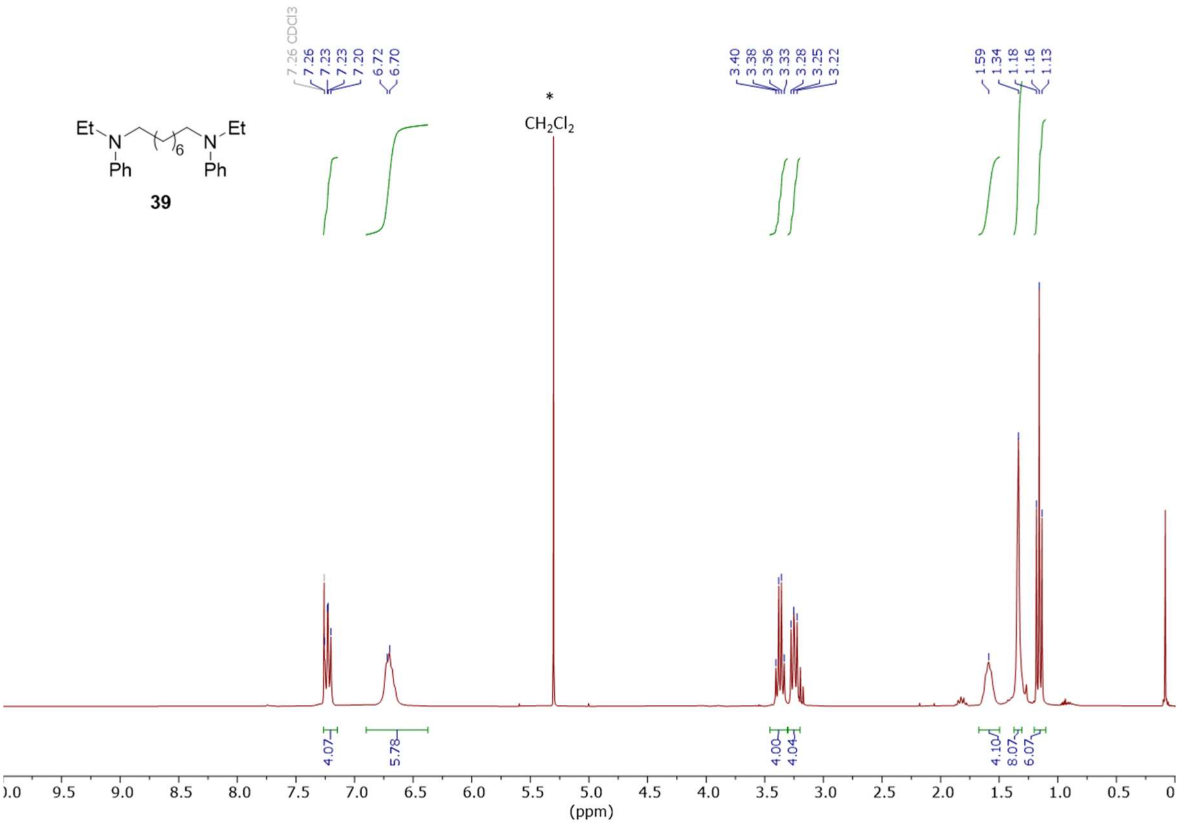

^{13}C NMR 101 MHz

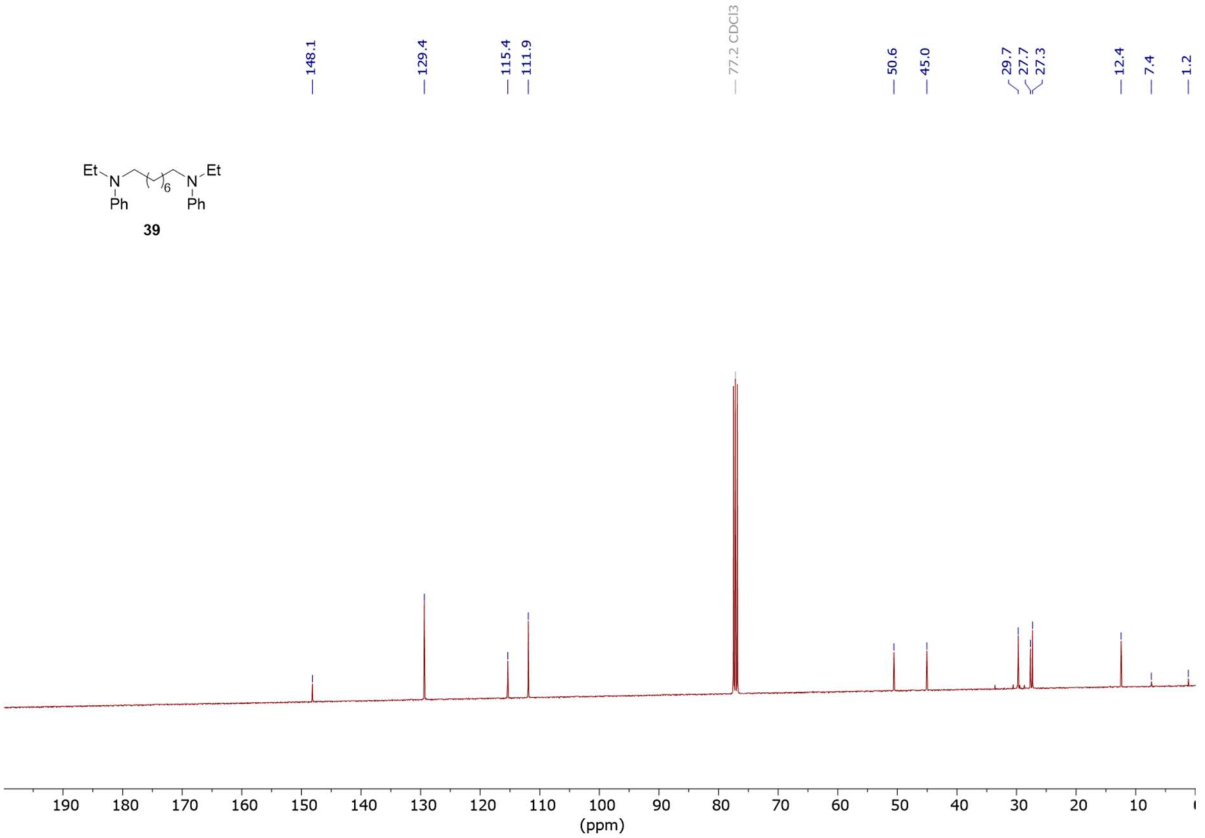
148.1
129.4
115.4
111.9
77.2 CDCl3
50.6
45.0
29.7
27.7
27.3
12.4
7.4
1.2
Et
N
6
Ph
39
190
180
170
160
150
140
130
120
110
100
90
80
70
60
50
40
30
20
10
(ppm)

DEPT

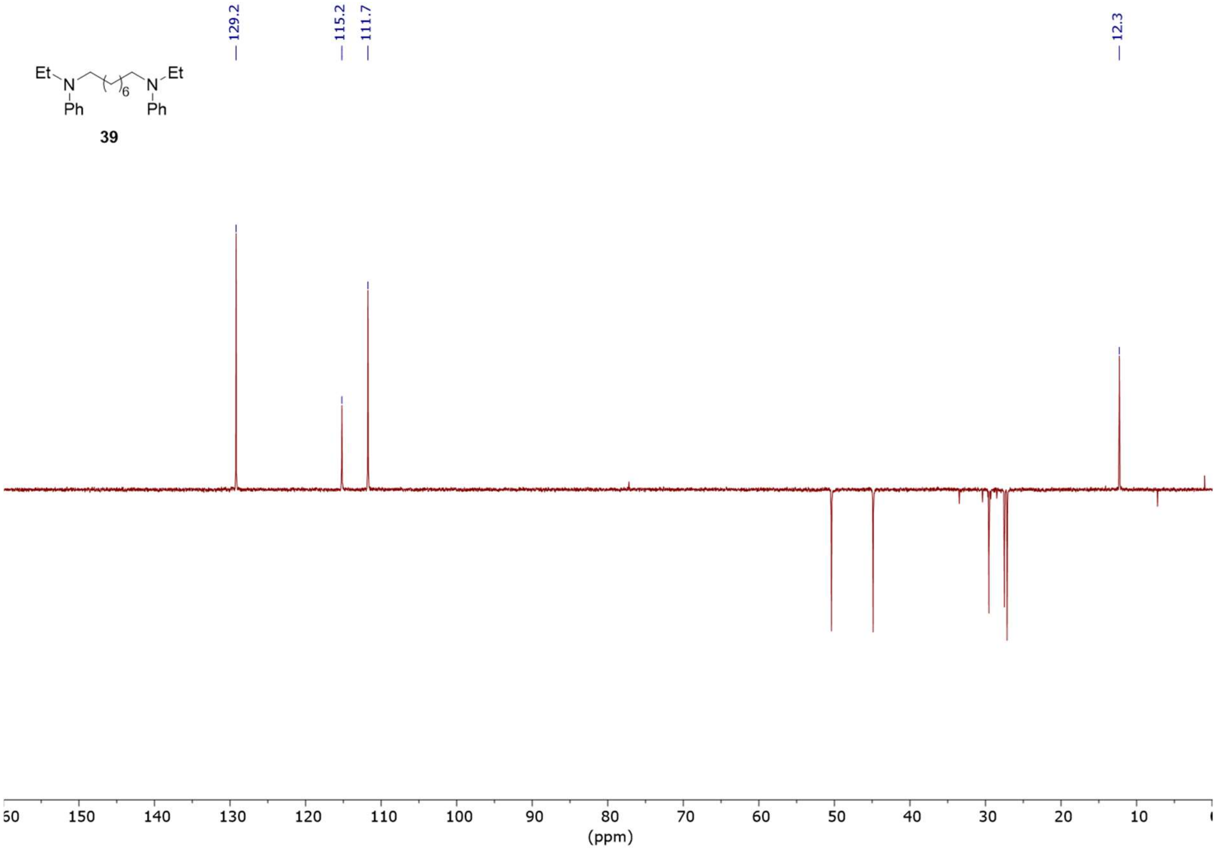
129.2
115.2
111.7
12.3
Et
N
6
Ph
39
150
140
130
120
110
100
90
80
70
60
50
40
30
20
10
(ppm)
